# Preventive Effects of Resistance Training on Hemodynamics and Kidney Mitochondrial Bioenergetic Function in Ovariectomized Rats

**DOI:** 10.3390/ijms26010266

**Published:** 2024-12-31

**Authors:** Anne L. F. Queiroz, Christopher B. Garcia, João P. M. O. Silva, Diego F. A. Cavalini, André V. Alexandrino, Anderson F. Cunha, Anibal E. Vercesi, Roger F. Castilho, Gilberto E. Shiguemoto

**Affiliations:** 1 Department of Physiological Sciences, Interinstitutional Post-Graduate Program of Physiological Sciences, Federal University of São Carlos (UFSCar), São Carlos 13.566-490, SP, Brazil; alfq06@gmail.com (A.L.F.Q.); crisbarzaque@gmail.com (C.B.G.); df.cavalini@gmail.com (D.F.A.C.); avalex@uol.com.br (A.V.A.); 2 Post-Graduate Program of Physiotherapy, Federal University of São Carlos (UFSCar), São Carlos 13.566-490, SP, Brazil; 3 Department of Genetics and Evolution, Federal University of São Carlos (UFSCar), São Carlos 13.566-490, SP, Brazil; joaopedro.maia7@gmail.com (J.P.M.O.S.); anderf@ufscar.br (A.F.C.); 4 Department of Biological Sicences, Central Paulista University Center (UNICEP), Campus São Carlos, São Carlos 13.570-300, SP, Brazil; 5 Department of Pathology, University of Campinas (UNICAMP), Campinas 13.083-970, SP, Brazil; anibal@unicamp.br (A.E.V.); rogerc@unicamp.br (R.F.C.)

**Keywords:** resistance training, kidney mitochondrial function, ETC, OXPHOS, ovariectomy, hemodynamic parameters

## Abstract

Menopause occurs due to the depletion of the ovarian reserve, leading to a progressive decline in estrogen (E2) levels. This decrease in E2 levels increases the risk of developing several diseases and can coexist with chronic kidney disease (CKD). Arterial hypertension (AH) is another condition associated with menopause and may either contribute to or result from CKD. Ovariectomy (OVX) induces hypoestrogenism, which can lead to mitochondrial bioenergetic dysfunction in the kidneys. Previous studies have suggested that exercise training has beneficial effects on adults with CKD and AH. To investigate the effects of OVX and resistance training (RT) on hemodynamic parameters and mitochondrial bioenergetic function of the kidney, female Wistar rats were divided into ovariectomized (OVX) and intact (INT) groups. These rats were either kept sedentary (SED) or subjected to RT for thirteen weeks. The RT involved climbing a vertical ladder with a workload apparatus. Hemodynamic parameters were assessed via tail plethysmography. Mitochondrial respiratory function was evaluated with high-resolution respirometry. Gene expression related to the electron transport chain (ETC) and oxidative phosphorylation (OXPHOS) was evaluated by real-time qPCR. At week 13, key hemodynamic parameters (systolic blood pressure and mean arterial pressure) were significantly elevated in the OVX-SED group. Compared with those in the other groups, mitochondrial bioenergetics were impaired in the OVX-SED group. In contrast, the trained groups presented improved mitochondrial bioenergetic function compared with the sedentary groups. OVX led to reduced gene expression related to the mitochondrial ETC and OXPHOS, whereas RT both prevented this reduction and increased gene expression in the trained groups. Our results indicate that hypoestrogenism significantly decreases OXPHOS and ETC capacity in the kidneys of sedentary animals. However, RT effectively increased the expression of genes related to mitochondrial ETC and OXPHOS, thereby counteracting the effects of OVX.

## 1. Introduction

Natural menopause is clinically diagnosed after 12 months of amenorrhea without a pathological cause [1]. Additionally, natural menopause is characterized by an increase in follicle-stimulating hormone (FSH) levels to >30 mIU/L and permanent cessation of menstruation due to the end of ovulation [2]. Clinical medicine has long recognized the link between menopause and the accelerated development of vascular diseases and osteoporosis, which remain significant health concerns for women [3]. Furthermore, menopause significantly increases the risk of cardiovascular disease (CVD) by more than threefold among women with normal kidney function [4]. Menopause is a known risk factor for CVD, primarily due to the detrimental effects of estrogen withdrawal on cardiovascular function and metabolism [5].

Numerous studies have demonstrated that early menopause, including both natural and surgical menopause, is associated with various diseases, such as CVD, diabetes, and osteoporosis [6,7,8]. The protective role of estrogen in maintaining cardiovascular health, bone density, and insulin sensitivity may be a significant factor in these associations [7,8]. Estrogen also appears to have a renoprotective effect in women [9], suggesting that early menopause may be linked to a greater risk of chronic kidney disease (CKD) [10]. Additionally, early menopause often occurs in women with CKD [2]. CKD is a prevalent health issue that affects approximately 13.4% of adults worldwide, with a higher incidence in women than in men [11]. This condition is associated with increased risks of mortality, cardiovascular problems, and progression to renal failure [12]. The presence of CKD comorbid with other conditions, such as type 2 diabetes mellitus and hypertension, can accelerate progression to end-stage renal disease, which is also known as stage V CKD. This negative outcome significantly increases the risk of cardiovascular morbidity and mortality, including ischemic heart disease, cerebrovascular disease, and peripheral vascular disease [13].

Hypertension is a major risk factor for cardiovascular and renal diseases [14]. The association between elevated blood pressure (BP) and CKD is complex because hypertension can be both a cause and a consequence of kidney dysfunction [15]. Owing to the reciprocal relationship between hypertension and CKD, individuals with hypertension are likely to experience a cycle of interconnected health issues [16]. Consequently, it can be inferred that individuals with hypertension are at greater risk of developing CKD than individuals without hypertension [17].

Bilateral ovariectomy (OVX) is the most widely studied animal model for investigating menopausal processes that closely resemble those in humans, thus making it the gold standard in preclinical research for evaluating the effects of gonadal hormones [18,19]. OVX is particularly effective at inducing key risk factors associated with metabolic syndrome [18]. Additionally, OVX can lead to the development of visceral obesity, atherogenic dyslipidemia, hepatic steatosis, and hypertension [20,21]. OVX rodents may exhibit complications similar to those experienced by menopausal women, including endothelial dysfunction, atherosclerotic lesions, osteoporosis, cognitive decline [16,22,23,24], and CKD [25]. Furthermore, OVX can eliminate the protective effects of estrogen in models of renal ischemia, while estrogen administration to aged female mice restores tolerance to ischemic injury [26]. Renoprotection by estrogen is also described in other forms of kidney injury, including rodent models of chronic allograft nephropathy, age-related glomerular damage, and hypertensive nephrosclerosis [27].

Owing to its high metabolic activity and abundance of mitochondria, the kidney relies on stable mitochondrial function to produce ATP for active tubular transport [28]. Mitochondria are crucial for various cellular processes, including biosynthesis, redox balance, calcium regulation, inflammation control, and cell death pathways. Consequently, mitochondria play essential roles in supporting the high-energy demands of the kidney, which is an organ that is rich in these vital organelles [29]. Mitochondrial dysfunction is central to the pathogenesis of kidney diseases and is linked to reduced mitochondrial DNA copy number, impaired membrane potential, enhanced mitochondrial oxidative stress, a significant decrease in mitochondrial biogenesis and ATP production, and impaired mitochondrial dynamics [27,30,31,32]. Mitochondria play an important role in estrogen’s effects on energy metabolism. Estrogens affect mitochondria in multiple aspects including protein content and activity, phospholipid content of membranes, oxidant and antioxidant capacities, oxidative phosphorylation (OXPHOS), and calcium retention capacities [33]. Reduction of estrogen or its receptor (ER-α and ER-β) is closely associated with impaired energy metabolism and mitochondrial dysfunction [34], which is increasingly recognized as an initiator of and contributor to acute kidney injury (AKI) and CKD [27].

Postmenopausal women have relied on hormone replacement therapy (HRT) for years to alleviate menopausal symptoms [35]. Nevertheless, the use of HRT remains controversial, as the prolonged use of HRT, including the use of synthetic hormones in specific combined therapies (e.g., estradiol hemihydrate and drospirenone or estradiol valerate and cyproterone acetate) has been associated with increased risks of breast cancer [36] and stroke [37]; on the other hand, estrogen alone or combined with progestogen replacement has been shown to result in a 50% reduction in heart disease, including heart failure, coronary events, and cardiovascular mortality [38]. Therefore, it is crucial to explore alternative therapeutic options for managing menopause, including complementary and alternative therapies [39]. Given the elevated risks of breast cancer and cardiovascular disease linked to HRT and the uncertain efficacy of alternative treatments, physical activity and exercise present promising alternatives for improving well-being during and after the menopausal transition [40].

Aerobic and resistance exercises are important components of comprehensive fitness programs, especially for menopausal women. Aerobic exercise has been widely shown to exert cardiovascular benefits, including reductions in arterial pressure (AP) and improvements in cardiac vagal tone and baroreflex sensitivity; these benefits have been observed in both ovariectomized hypertensive rats and postmenopausal women [41]. Conversely, resistance training (RT) is important for enhancing muscle strength, which alleviates menopause-related bone, skeletal muscle and blood pressure issues in both ovariectomized rats and menopausal women [22,24,42]. In non-CKD patients, aerobic and resistance exercise has positive effects on inflammatory cytokines, insulin resistance, obesity, cardiovascular risk factors, microalbuminuria, and anemia associated with chronic disease [43].

In the context of renal diseases, the global impact of different programs of exercise training on the prevention and management has yet to be fully understood [44]. Renoprotective effects of exercise training have been demonstrated in both studies with pre-conditioning progressive aerobic exercise in experimental models of AKI [45] and CKD [46], as well as in studies with exercise training conducted after the establishment of acute [47] and chronic renal injuries [48]. The precise ways in which exercise training provides renoprotective effects remain uncertain, but extensive evidence indicates that consistent aerobic exercise regimens offer notable anti-inflammatory [45], anti-fibrotic [49], antioxidant [50], and anti-apoptotic [51] effects. Endurance exercise has been shown to increase mitochondrial biogenesis, and sirtuin 1 (SIRT1) content and activity [52]. SIRT 1 can protect kidney tubular cells function [53], and as a humoral agent, can have a strong renoprotective effect against ischemic injury or toxic substance [54]. However, the potential benefits of exercise training, especially resistance exercise, for patients with renal disease remain unclear, particularly concerning mitochondrial respiratory function and OXPHOS.

In view of the arguments highlighted above, the main objective of this work was to investigate the effects of OVX and RT on hemodynamic parameters and mitochondrial bioenergetic function of the kidney in female rats. Our primary hypothesis is that OVX may lead to adverse changes in hemodynamic parameters and mitochondrial bioenergetic function, as well as alterations in the expression of genes involved in the electron transport chain (ETC) and OXPHOS of the kidney. Additionally, we hypothesized that RT could mitigate or prevent these deleterious changes.

## 2. Results

### 2.1. Body Parameters, Body Mass and Tissue Mass

At the beginning of the study, the experimental groups showed no significant differences in body mass (BM), indicating homogeneity among the sample. At the end of the experiment, OVX significantly altered the BM in both the OVX-SED and OVX-RT groups compared with the INT-SED (*p* = 0.020) and INT-RT groups (*p* = 0.001). Despite physical training, there were no significant differences in BM between the OVX-SED and OVX-RT groups. However, the INT-RT group presented a lower BM than the INT-SED group (*p* = 0.04), thus highlighting the effectiveness of resistance training in controlling the BM of intact rats. Compared with the initial measurements, all of the groups presented increased BM at the end of the experimental period (*p* < 0.05), as shown in Table 1.

The uterine mass-to-tibia length (U/T) ratio was used to assess the efficacy of OVX (Table 1). A reduction in the U/T ratio was expected in all OVX groups, indicating decreased ovarian hormone production. The results confirmed the success of the OVX and revealed a statistically significant reduction in the uterine mass in the OVX groups compared with the INT groups (*p* = 0.001). No significant differences were observed in the left kidney mass among the groups.

### 2.2. Maximal Workload

**Figure 1:**
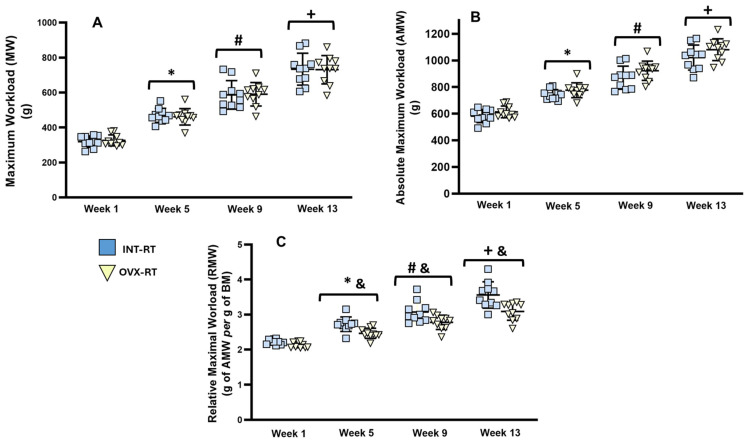
Evaluation of Maximum Workload. Experimental trained groups: INT-RT (intact resistance training) and OVX-RT (ovariectomized resistance training). (**A**) Maximum workload (grams); (**B**) absolute maximal workload (grams); (**C**) relative maximum workload (grams of AMW per gram of BM). Evaluation periods: weeks, 1, 5, 9, and 13. Values are expressed as means ± standard deviations (SD), with n = 10 per group. To compare differences in maximal workloads (MW, AMW and RMW) between groups in the same week, and between weeks (time) in the same group, two-way ANOVA and post hoc Sidak’s multiple comparisons test were used, *p* < 0.05. * indicates a significant difference between week 5 and week 1; # indicates a significant difference between week 9 and week 5; + indicates a significant difference between week 13 and week 9; & indicates a significant difference between groups in the same week.

Figure 1 displays the maximal workload (MW, Figure 1A), absolute maximal workload (AMW, Figure 1B), and relative maximal workload (RMW, Figure 1C) values for the trained groups (INT-RT and OVX-RT). Both trained groups exhibited gradual and similar increases in the MW and AMW from week 1 to week 13; therefore, no interaction between RT and OVX was revealed to be statistically significant by two-way ANOVA (F_(1,18)_ = 0.5620, *p* = 0.4631). The OVX-RT group presented an average increase of 124.05% in the MW from week 1 to week 13 (*p* < 0.0001, Figure 1A), whereas the INT-RT group presented an average increase of 129.73% in the MW over the same period (*p* < 0.0001, Figure 1A). Differences in BM between the trained groups from week 5 to week 13 were significant in the RMW between the trained groups (INT and OVX), and the two-way ANOVA revealed an interaction between RT and OVX in week 5 (F_(1,18)_ = 4.866, *p* = 0.0406) and week 13 (F_(1,18)_ = 5.267, *p* = 0.0340) (Figure 1C).

### 2.3. Hemodynamic Parameters: Systolic Blood Pressure, Diastolic Blood Pressure, Mean Arterial Pressure, Heart Rate, and Double Product

**Figure 2:**
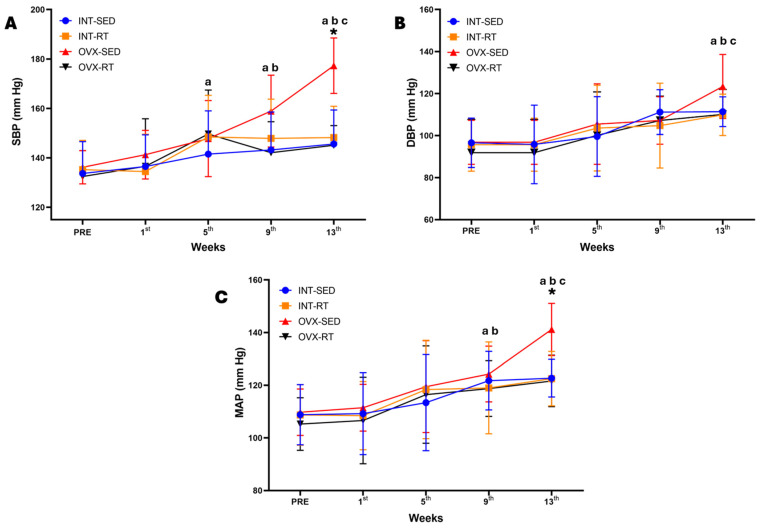
Temporal hemodynamics evaluation over 13 weeks of the experimental protocol. Values are expressed as the mean ± standard deviation (SD), n = 10 per group. (**A**) SBP: systolic blood pressure; (**B**) DBP: diastolic blood pressure; (**C**) MAP: mean arterial pressure. Intact group (INT): intact sedentary (INT-SED) and intact resistance training (INT-RT). Ovariectomy group (OVX): ovariectomy sedentary (OVX-SED) and ovariectomy resistance training (OVX-RT). To compare differences in hemodynamics parameters (SBP, DBP, and MAP) between groups in the same week, and between weeks (time) in the same group, two-way ANOVA and post hoc Sidak’s multiple comparisons test were used, *p* < 0.05. * denotes a significant difference compared with the other groups; ^a^ denotes a significant difference compared with the initial evaluation (PRE); ^b^ denotes a significant difference compared with week 5; ^c^ denotes a significant difference compared with week 9.

Figure 2 presents the hemodynamic assessments conducted over the 13 weeks of the experimental protocol. Systolic blood pressure (SBP, Figure 2A) was significantly greater in the OVX-SED group (177.30 mmHg) than in the other groups only at the 13th week, including the INT-SED (145.60 mmHg, *p* < 0.0001), INT-RT (148.22 mmHg, *p* < 0.0001), and OVX-RT groups (145.11, *p* < 0.0001), and also demonstrated an interaction between time and groups in SBP at this moment of evaluation (F_(3,36)_ = 4.884, *p* = 0.0060). Notably, the increase in SBP in the OVX-SED group began at the 9th week (SBP = 158.90 mmHg) compared with the initial evaluation (PRE) (136.20 mmHg, *p* = 0.0003), and the SBP continued to increase significantly at the 9th and 13th weeks compared with the previous assessments. A similar pattern was observed for the mean arterial pressure (MAP) in the OVX-SED group (Figure 2C), which showed a significant increase in MAP (141.19 mmHg) only at the 13th week compared with the other groups, including the INT-SED (122.69 mmHg, *p* = 0.0015), INT-RT (120.46 mmHg, *p* = 0.0014), and OVX-RT groups (121.66, *p* = 0.0007), and also demonstrating an interaction between time and groups in MAP at this moment of evaluation (F_(3,36)_ = 3.809, *p* = 0.0181). The MAP for the OVX-SED group significantly increased at the 9th week (MBP = 124.26 mmHg) compared with the initial evaluation (PRE) (109.80 mmHg, *p* = 0.0036) and continued to increase at the 13th week (141.18 mmHg, *p* < 0.0000). The OVX-SED group presented a significant increase in diastolic blood pressure (DBP) only at the 13th week (123.40 mmHg) compared with the 9th week (107.20 mmHg, *p* = 0.0063) and the other previous weeks (Figure 2B).

The double product (DP), which is an indicator of cardiac effort, may reflect significant myocardial overload. This parameter is indirectly measured by multiplying the SBP by the heart rate (HR). Our study revealed that among OVX-SED rats, the DP significantly increased from week 9 onward (DP = 70,406.44) compared with the INT-SED (DP = 59,868.70, *p* = 0.0391), INT-RT (DP = 58,717.56, *p* = 0.0172), and OVX-RT (DP = 57,919.56, *p* = 0.0094) groups (Table 2. The variation in SBP was the main factor responsible for the change in the DP. In the OVX-RT group, there was no significant increase in the DP. The final DP values in the OVX-RT group were similar to those in the INT-SED and INT-RT groups, suggesting that resistance training may indeed lead to beneficial cardiac remodeling in terms of hemodynamic responses. Table 2 presents the SBP, HR, and DP results for the experimental rats.

### 2.4. Mitochondrial Respiratory Function

**Figure 3:**
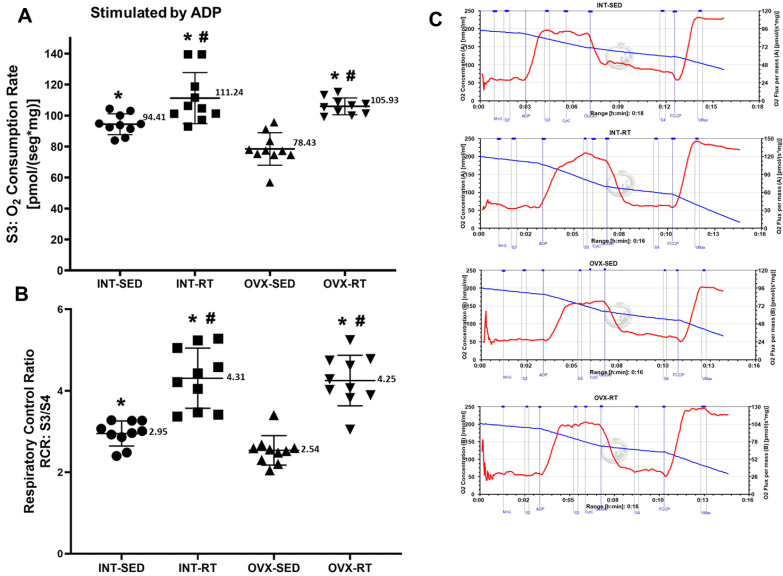
High-resolution respirometry performed on kidney cortex biopsies. (**A**) State 3 (S3), oxygen consumption during maximal ADP-stimulated respiration supported by complex I substrates. (**B**) Respiratory acceptor control ratio (RCR), determined by S3/S4 ratio. (**C**) Representative traces for all groups. Values are expressed as means ± standard deviations (SD), n = 10 per group. Groups include: intact (INT), intact sedentary (INT-SED) and intact resistance training (INT-RT); and ovariectomy (OVX), ovariectomy sedentary (OVX-SED) and ovariectomy resistance training (OVX-RT). Statistical analyses were performed using one-way ANOVA and Fisher’s post hoc test, with significance set at *p* < 0.05. * denotes a significant difference for the OVX-SED group; # denotes a significant difference for the INT-SED group.

An important finding of the present study was the significant decrease in ADP-stimulated oxygen consumption (respiratory state 3; OXPHOS capacity) by kidney tissue (78.43 pmol/[s·mg]) supported by complex I substrates (malate and glutamate) as a result of OVX in the sedentary group (OVX-SED) compared with the INT-SED (94.41 pmol/[smg], *p* = 0.0019), INT-RT (111.24 pmol/[s·mg], *p* < 0.0001), and OVX-RT groups (105.93 pmol/[s·mg], *p* < 0.0001) (Figure 3A). The reduction in OXPHOS capacity in the OVX-SED group was 16.92% relative to the INT-SED group. Compared with the sedentary groups, the trained groups (INT-RT and OVX-RT) presented greater OXPHOS capacity: INT-RT versus INT-SED (17.83%), INT-RT versus OVX-SED (41.83%), OVX-RT versus OVX-SED (35.06%), and OVX-RT versus INT-SED (12.20%). For the respiratory acceptor control ratio (RCR = S3/S4), lower values were observed in the OVX-SED group (2.54) than in the INT-SED group (2.95, *p* = 0.0130) and the trained groups (OVX-RT, 4.25 and INT-RT, 4.31, *p* < 0.0001). Compared with the INT-SED group, the trained groups (INT-RT and OVX-RT) had significantly greater RCR values (*p* < 0.0001 for both) (Figure 3B). Representative traces for all groups can be seen in Figure 3C.

**Figure 4:**
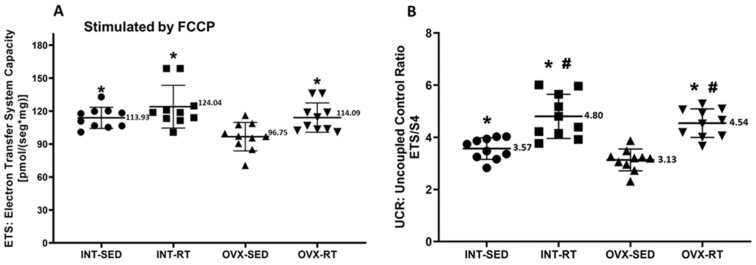
Mitochondrial noncoupled respiration in kidney cortex biopsy. Panel (**A**) electron transfer system capacity (ETS), a noncoupled state measured under FCCP stimulation. Panel (**B**) presents the uncoupled control ratio (UCR), calculated as ETS divided by respiratory state 4. Results are expressed as means ± standard deviations (SD), with n = 10 per group. The study includes the groups: ovariectomy (OVX), subdivided into sedentary ovariectomy (OVX-SED) and resistance training ovariectomy (OVX-RT), and intact (INT), subdivided into sedentary intact (INT-SED) and resistance training intact (INT-RT). Statistical significance was determined using one-way ANOVA and Fisher’s post hoc test, with a threshold of *p* < 0.05. * denotes a significant difference for the OVX-SED group, while # denotes a significant increase compared with the INT-SED group.

In terms of ETS capacity (experimental oxygen consumption rate stimulated by FCCP), a significant decrease in oxygen consumption was observed in the OVX-SED group (96.75 pmol/[s·mg]) compared with the INT-SED group (113.93 pmol/[s·mg], *p* = 0.0107) and the trained groups (INT-RT, 124.04 pmol/[s·mg], *p* = 0.0001; OVX-RT, 114.09 pmol/[s·mg], *p* = 0.0101) (Figure 4A). For the UCR (uncoupled control ratio), the OVX-SED group presented lower values (3.13) than did the INT-SED group (3.57, *p* = 0.0312) and the trained groups (OVX-RT, 4.54; INT-RT, 4.80, *p* < 0.0001). Compared with the INT-SED group, the trained groups (INT-RT and OVX-RT) presented significantly greater UCR values (*p* = 0.000033 and *p* = 0.000619, respectively) (Figure 4B).

**Figure 5:**
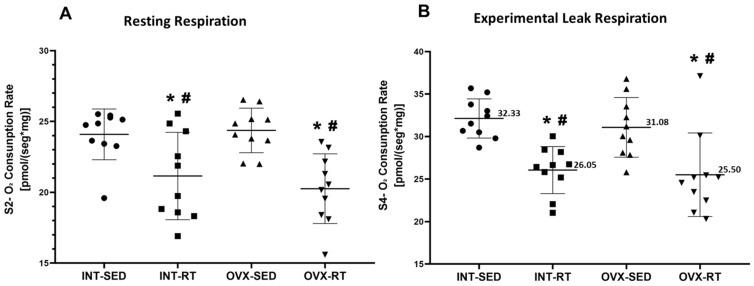
High-Resolution Respirometry of Kidney Cortex Biopsies. (**A**) State 2 (S2): Resting or routine respiration; (**B**) State 4 (S4): Oligomycin-inhibited state or experimental leak respiration. Values are presented as means ± standard deviations (SD), n = 10 per group. The intact group (INT) includes the sedentary intact (INT-SED) and resistance training intact (INT-RT) subgroups. The ovariectomy group (OVX) includes the sedentary ovariectomy (OVX-SED) and resistance training ovariectomy (OVX-RT) subgroups. Statistical analysis was performed using one-way ANOVA followed by Fisher’s post hoc test, with significance set at *p* < 0.05. * denotes a significant difference compared with the OVX-SED group; # denotes a significant difference compared with the INT-SED group.

In state 2 (S2) or resting respiration, the OVX-SED group exhibited significantly greater oxygen consumption (24.37 pmol/[s·mg]) than both the INT-RT (21.15 pmol/[s·mg], *p* = 0.0035) and OVX-RT groups (20.25 mol/[s·mg], *p* = 0.0003) (Figure 5A). Similarly, the INT-SED group presented elevated oxygen consumption (24.09 pmol/[s·mg]) compared with the INT-RT (21.15 pmol/[s·mg], *p* = 0.0072) and OVX-RT groups (20.25 mol/[s·mg], *p* = 0.0006) (Figure 5A). No significant differences in S2 oxygen consumption were observed between the sedentary groups (INT-SED and OVX-SED).

In state 4 (S4), the oligomycin-inhibited state or experimental leak respiration, the OVX-SED group presented a significant increase in oxygen consumption (31.08 pmol/[s·mg]) compared with both the INT-RT (26.05 pmol/[s·mg], *p* = 0.0029) and OVX-RT groups (25.50 mol/[s·mg], *p* = 0.0011) (Figure 5B). Similarly, the INT-SED group had elevated oxygen consumption (32.33 pmol/[s·mg]) compared with the INT-RT (26.05 pmol/[s·mg], *p* = 0.0004) and OVX-RT groups (25.50 mol/[s·mg], *p* = 0.0001) (Figure 5B). No significant differences in S4 oxygen consumption were found between the sedentary INT and OVX groups.

### 2.5. Citrate Synthase Activity and mRNA Gene Expression of Citrate Synthase

**Figure 6:**
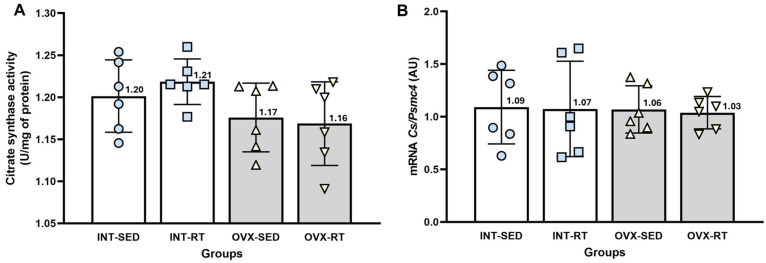
Analyses of citrate synthase. (**A**) Citrate synthase activity in kidney cortex biopsies. (**B**) Real-time PCR analysis of citrate synthase gene expression, normalized to *Psmc4*. Values are presented as means ± standard deviations (SD). The intact group (INT) includes the intact sedentary (INT-SED) and intact resistance training (INT-RT) subgroups. The ovariectomy group (OVX) comprises the ovariectomy sedentary (OVX-SED) and ovariectomy resistance training (OVX-RT) subgroups. No significant differences were observed between groups, as determined by one-way ANOVA and Fisher’s post hoc test, *p* < 0.05.

No significant differences were observed between groups in terms of citrate synthase activity (Figure 6A). Citrate synthase is a well-established marker of mitochondrial mass in skeletal muscle [42] and can serve as a reliable indicator of mitochondrial mass in kidney tissue. Similarly, the mRNA gene expression of citrate synthase did not significantly differ across the groups, further supporting the findings from the citrate synthase activity measurements (Figure 6B).

### 2.6. Quantification of mRNA Gene Expression Related to Mitochondrial ETC and OXPHOS

#### 2.6.1. Gene Expression of ETC Protein Complexes (I to IV)

**Figure 7:**
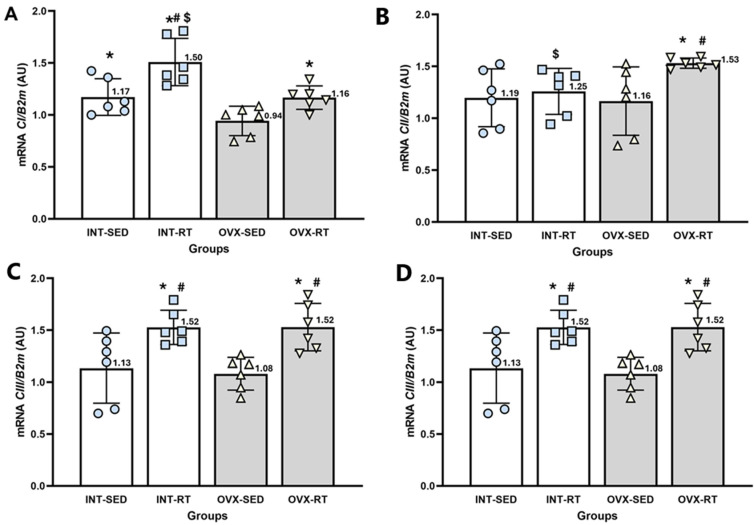
RT–qPCR analysis of gene expression related to the electron transport chain complexes I to IV in the kidney cortex. *B2m* (beta-2 microglobulin) was used as an endogenous reference gene. (**A**) *CI*: NADH dehydrogenase; (**B**) *CII:* succinate dehydrogenase; (**C**) *CIII*: ubiquinone cytochrome c oxidoreductase; and (**D**) *CIV*: cytochrome c oxidase. Expression levels are reported in arbitrary units (AU). Groups include INT-SED (intact sedentary), INT-RT (intact resistance training), OVX-SED (ovariectomy sedentary), and OVX-RT (ovariectomized resistance training). Values are expressed as the mean ± standard deviation (SD). Symbols above the SD bars indicate statistical differences between groups, determined by one-way ANOVA followed by Fisher’s post hoc test, *p* < 0.05. * denotes a significant difference compared with the OVX-SED group; # denotes a significant increase compared with the INT-SED group; and $ denotes a significant difference compared with the OVX-RT group.

NADH dehydrogenase (Complex I [*CI*], Figure 7A) plays a crucial role in transferring electrons into the electron transport chain and pumping H^+^ into the intermembrane space. The gene expression levels of Complex I were significantly lower in the OVX-SED group than in the INT-SED group (*p* = 0.020). Both resistance-trained groups (OVX-RT and INT-RT) presented significantly greater expression of Complex I than did the OVX-SED group (*p* = 0.044 and *p* = 0.0001, respectively). Moreover, the INT-RT group presented higher expression levels than the other groups did, including the OVX-SED (*p* = 0.0001), OVX-RT (*p* = 0.0001) and INT-SED (*p* = 0.001) groups.

Succinate dehydrogenase (Complex II [*CII*], Figure 7B) is involved in transferring electrons into the electron transport chain. The gene expression levels of Complex II in the OVX-SED group did not differ significantly from those in the INT-SED group. However, the INT-RT group displayed significantly lower expression than the OVX-RT group did (*p* = 0.012). In contrast, the OVX-RT group presented significantly higher expression levels than both the OVX-SED (*p* = 0.015) and INT-SED (*p* = 0.025) groups.

The gene expression of ubiquinone: cytochrome c oxidoreductase (Complex III [*CIII*], Figure 7C), did not significantly differ between the OVX-SED and INT-SED groups. However, both trained groups (OVX-RT and INT-RT) presented significantly higher expression levels of this complex than the OVX-SED group (*p* = 0.0001 and *p* = 0.001, respectively) and the INT-SED group (*p* = 0.002 and *p* = 0.004, respectively).

Cytochrome c oxidase (Complex IV [*CIV*], Figure 7D) is responsible for accepting electrons, converting molecular oxygen and protons into H_2_O, and pumping H^+^ into the intermembrane space. Compared with the INT-SED group, the OVX-SED group presented significantly lower expression levels of cytochrome c oxidase (*p* = 0.0001). Additionally, both trained groups (OVX-RT and INT-RT) presented significantly greater expression of cytochrome c oxidase than the OVX-SED (*p* = 0.0001 for both) and INT-SED groups (*p* = 0.022 for OVX-RT and *p* = 0.013 for INT-RT).

#### 2.6.2. Gene Expression of the Phosphorylation System (Atps, Pic, and Ant1) and Ucp2

**Figure 8:**
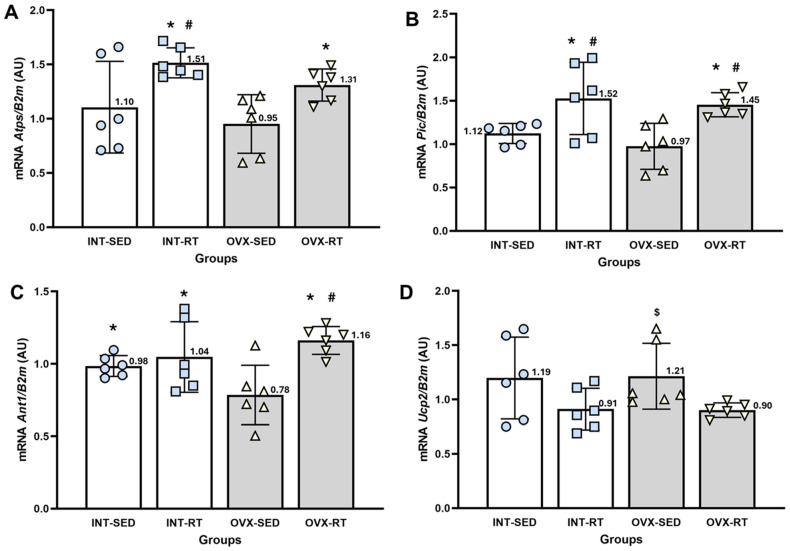
RT–qPCR analysis of gene expression related to the OXPHOS system in the kidney cortex. *B2m* (beta-2 microglobulin) was used as an endogenous reference gene. (**A**) *Atps*: ATP synthase; (**B**) *Pic*: phosphate carrier; (**C**) *Ant1*: adenine nucleotide translocase; and (**D**) *Ucp2*: uncoupling protein 2. Expression levels are reported in arbitrary units (AU). Groups include INT-SED (intact sedentary), INT-RT, (intact resistance training), OVX-SED (sedentary ovariectomy), and OVX-RT (ovariectomized resistance training). Values are expressed as the mean ± standard deviation (SD). Symbols above the SD bars indicate statistical differences between groups, determined by one-way ANOVA followed by Fisher’s post hoc test, *p* < 0.05. * denotes a significant difference compared with the OVX-SED group; # indicates a significant difference compared with the INT-SED group; $ denotes a significant increase compared with the OVX-RT group.

ATP synthase (Atps, Figure 8A) is responsible for phosphorylating ADP into ATP. The *Atps* expression levels in the OVX-SED group did not differ significantly from those in the INT-SED group. However, both trained groups (OVX-RT and INT-RT) presented significantly greater *Atps* expression than did the OVX-SED group (*p* = 0.031 and *p* = 0.001, respectively). Additionally, the INT-RT group presented greater expression than the INT-SED group did (*p* = 0.015).

The phosphate carrier (Pic, Figure 8B) promotes the transport of inorganic phosphate molecules and H^+^ from the intermembrane space to the mitochondrial matrix. The *Pic* expression levels in the OVX-SED group did not differ significantly from those in the INT-SED group. However, both trained groups (OVX-RT and INT-RT) presented significantly greater Pic expression than the OVX-SED (*p* = 0.002 and *p* = 0.0001, respectively) and INT-SED groups (*p* = 0.021 and *p* = 0.006, respectively).

The gene expression of adenine nucleotide translocase (Ant1, Figure 8C), which promotes the exchange of ATP and ADP between the mitochondrial matrix and the intermembrane space, was significantly lower in the OVX-SED group than in the INT-SED group (*p* = 0.034). Both trained groups (OVX-RT and INT-RT) presented significantly greater Ant1 expression than the OVX-SED group did (*p* = 0.0001 and *p* = 0.011, respectively). Additionally, the OVX-RT group presented significantly greater expression than the INT-SED group did (*p* = 0.0001).

Uncoupling protein 2 (Ucp2, Figure 8D) is responsible for the leakage of protons from the intermembrane space to the mitochondrial matrix. The *Ucp2* expression levels in the OVX-SED group did not differ significantly from those in the INT-SED group. However, the OVX-SED group presented significantly greater expression than the OVX-RT group (*p* = 0.031).

**Figure 9:**
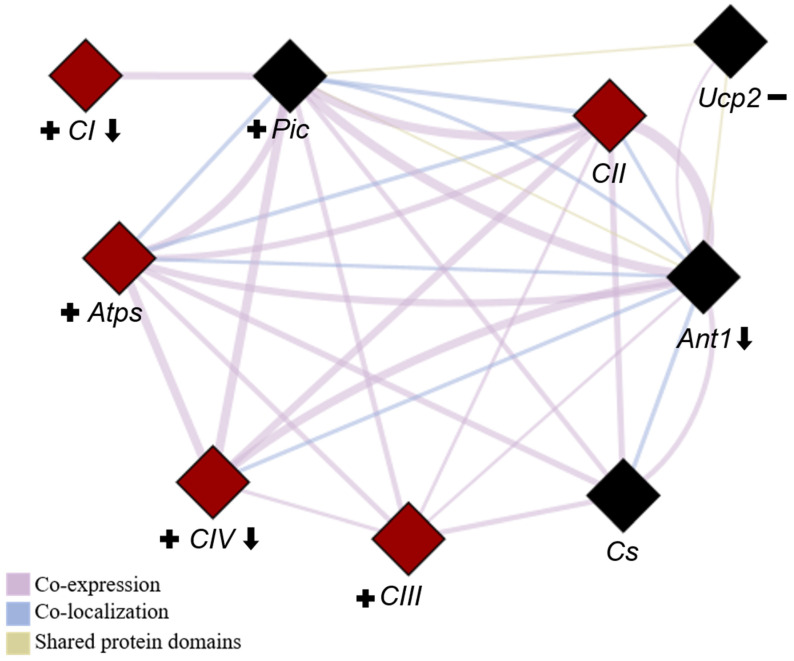
Network depicting the interactions among all evaluated genes. The network was generated using the GeneMANIA plugin of Cytoscape software (version 3.10.3) to identify all of the correlations among the genes presented in this study. Genes involved in respiratory chain complexes are highlighted in red, while those related to OXPHOS are represented by diamond shapes. *CI* (complex I), *CII* (complex II), *CIII* (complex III), *CIV* (complex IV), *Atps* (ATP synthase), *Ant1* (adenine nucleotide translocase-1), *Pic* (phosphate carrier), *Ucp2* (uncoupling protein) and *Cs* (citrate synthase). Downward arrows indicate genes negatively affected by ovariectomy (OVX), plus signs denote genes with increased expression due to resistance training (RT), and minus signs represent genes with reduced expression in response to RT.

To better illustrate the relationships between these genes, a network diagram was created to highlight key biological processes involving the genes (Figure 9). For clarity, we present the names of the complexes rather than the individual genes selected to represent them.

## 3. Discussion

The present study reveals four main findings: (i) ovariectomy promotes a decrease in OXPHOS capacity in kidney cortex biopsies of the sedentary group, which can be prevented by the resistance training (RT) protocol; (ii) OVX increases hemodynamics parameters—mainly SBP, DBP, MAP, and DP—in the sedentary group, which can also be prevented by the RT protocol; (iii) RT improves mitochondrial ETC and OXPHOS gene expression in the OVX animals; and (iv) kidney cortex biopsy is suitable for in situ analysis of the mitochondrial bioenergetics dysfunction in ovariectomized female rats.

The main hypothesis of our study, that OVX, the gold standard model of human menopause or hypoestrogenism [18], promotes negative changes in mitochondrial respiratory function and OXPHOS, as well as changes in the expression of genes related to the electron transport chain in the kidney cortex, was confirmed. Compared with the trained groups (INT-RT and OVX-RT), the ovariectomy sedentary group (OVX-SED) presented low oxygen consumption rates in the coupled respiratory state (S3, stimulated by ADP) or OXPHOS capacity and a decrease in the expression of genes (*CI*, *CII*, and *Ant1*) related to the ETC and OXPHOS. All of these deleterious changes promoted by the lack of estrogen were minimized or prevented by RT.

17β-estradiol (E2) acts centrally and systemically to regulate energy balance and metabolism [55]. Menopause occurs due to ovarian reserve depletion that results in a progressive decline in E2 levels [56]. This decrease in E2 levels promotes mitochondrial bioenergetic dysfunction through a decrease in complex I activity, as observed in the mRNA levels of complex I (*CI*), electron transfer, and OXPHOS responsiveness [42]. A reduction in OXPHOS capacity promoted by a decrease in E2 levels was observed in the OVX-SED group herein, which presented a reduction of 16.92% compared with the control sedentary group (INT-SED). This reduction was prevented and improved by RT, as the OVX-RT group presented superior OXPHOS capacity compared with the OVX-SED (35.06%) and INT-SED groups (12.20%). Compared with the INT-SED and OVX-SED groups, the INT-RT group presented the highest OXPHOS capacity, which was 16.92% and 41.83% greater than the capacities of the INT-SED and OVX-SED groups, respectively. Consistent with the reduction in OXPHOS capacity promoted by the absence of E2, the respiratory acceptor control ratio (RCR = S3/S2) was lower in the OVX-SED group than in all of the other groups: INT-SED, INT-RT, and OVX-RT. The RCR is the main indicator of mitochondrial respiratory function, reflecting the ability of mitochondria to switch between low and high respiratory rates in response to ADP, and has been suggested to be a good indicator for assessing mitochondrial respiratory dysfunction, even when the electron transport chain capacity remains intact [57,58]. Low RCR ratios can also be interpreted as a limited mitochondrial OXPHOS capacity [59].

The maximal capacity of the electron transport system (ETS capacity) is an open-circuit state established experimentally by complete uncoupling (noncoupled state) and can be estimated by promoting protonophore- or uncoupler-induced maximal oxygen consumption rate (maxOCR), which potentially exceeds the OXPHOS capacity [60,61]. In our study, ovariectomy reduced the ETS capacity and the uncoupling control ratio (UCR) of the kidney cortex biopsy in the sedentary group (OVX-SED) compared with those in the INT-SED group. A recent study in a model of nephrectomized male rats revealed a decrease in mitochondrial OXPHOS and ETS capacity in the kidney cortex, indicating that CKD induced by nephrectomy is a result of mitochondrial respiratory dysfunction [62]. The reduced ETS capacity in the OVX-SED group was prevented by the resistance training protocol used in our study, as the OVX-RT group presented higher values of ETS capacity and UCR; importantly, the ETS capacity was also improved in the intact trained group (INT-RT), which presented higher values of ETS capacity and UCR than the INT-SED group did. To our knowledge, our work is the first to present results showing a decrease in the OXPHOS and ETS capacity of kidney cortex biopsies from OVX sedentary female rats. In relation to the exercise training protocols, similar results showing improvements in OXPHOS and ETS capacity in OVX rats could be found only in muscle biopsies of the vastus lateralis of OVX rats subjected to RT [42] and in humans who performed RT [63], endurance (ET) and strength training (ST) [64].

In terms of resting mitochondrial respiration levels, state 2 (S2, physiological) and state 4 (S4, experimental) provide different states for estimating leak respiration [59]. In state 2, mitochondrial respiration remains slow, mainly compensating for passive proton leak and inner membrane ion channels such as uncoupling proteins or the permeability transition pore [65]. In state 4, mitochondrial respiration returns to a resting level caused by the presence of an ATP synthase inhibitor (oligomycin) [66]. Our study revealed high values of oxygen consumption in states 2 and 4 of the sedentary groups, OVX-SED and INT-SED, compared with both trained groups (OVX-RT and INT-RT), which presented low values. Proton leak is a physiological process that permits mitochondrial oxygen consumption to be carried out in the absence of OXPHOS, thus enabling certain cell types to generate heat, maintain carbon flux, and alter nutrient responses in the setting of specific metabolic demands [66]. Proton leak can also be pathological and compromise mitochondrial efficiency through the uncoupling of respiration from OXPHOS, thereby negatively affecting ATP production [67]. In the kidney, UCP-2 is involved in proton leak across the inner mitochondrial membrane [68]. Friederich et al. [69] reported that an increase in Ucp-2 mRNA expression resulted in mitochondrial uncoupling and increased oxygen consumption in the renal proximal tubular cells of diabetic rats. In our study, *Ucp-2* expression was greater in both sedentary groups (INT-SED and OVX-SED) than in the trained groups. Importantly, the elevated values of oxygen consumption in S2 and S4 (resting leak respiration) of the sedentary groups (OVX-SED and INT-SED) in our study were the main factors responsible for the low RCR and UCR values.

It is important to highlight that the kidney is one of the most energy-demanding organs in the human body [70] and has the second highest mitochondrial content and oxygen consumption after the heart [71]. The resting metabolic rate for the kidney is high because the kidney requires an abundance of mitochondria to provide sufficient energy to enable it to remove waste from the blood, reabsorb nutrients, regulate the balance of electrolytes and fluid, maintain acid–base homeostasis, and regulate blood pressure [70]. Mitochondrial dysfunction has been implicated in several forms of kidney disease [72], including diabetic nephropathy [73], chronic and acute kidney disease [74], and hypertensive kidney disease [75]. Therefore, evaluating renal respiratory parameters in detail is important for determining the nature of dysfunction. Each parameter, including basal respiration, ATP-linked respiration, maximal respiration, reserve capacity, and proton leak, can provide information about various functional aspects of the mitochondrion and the cell [76]. Mitochondria rely on the respiratory chain to produce ATP [70], which closely links the normal function of the kidneys to the respiratory chain [77]. In cases of renal failure, defects in the mitochondrial respiratory chain lead to disorders in ATP production, necessitating metabolic reprogramming of renal cells to adapt to injury and maintain the ATP supply [78,79].

Normalizing by citrate synthase (CS) activity is essential for adjusting results based on mitochondrial content [42]. The activities of the mitochondrial electron transfer chain (ETS, I to IV) are normalized by CS activity, which is a common indicator of mitochondrial content or abundance in muscle tissue [80,81], or to normalize oxidative and bioenergetic capacity in muscle [82] and kidney tissue [62]. Importantly, specifically in the kidney, increased CS activity is associated with increased circulating aldosterone levels, thus contributing to progressive renal injury [83] and to cisplatin-induced AKI and CKD progression [84]. In our study, CS activity and *Cs* expression in the renal cortex did not differ between groups, thus demonstrating that neither OVX nor the RT exercise protocol interfered with mitochondrial content, oxygen consumption or OXPHOS capacity in any of the mitochondrial respiratory states analyzed herein.

Women account for a substantial proportion of patients with CKD, which is often diagnosed at perimenopause or menopause [85]. Both CKD and menopause exhibit hormonal fluctuations that have a direct impact on cardiovascular health [86]. Among the major adverse outcomes related to CKD, CVD is one of the most important because it is the leading cause of death in this clinical population [87]. Importantly, the control of arterial hypertension is essential among CKD patients, as high blood pressure is a strong determinant of poor cardiovascular and renal outcomes [88]. In our study, the OVX-SED group presented high hemodynamic values compared with the other groups at the 13th week of evaluation, including SBP, MAP, and DP. Altogether, these values together may characterize a condition of arterial hypertension (AH) promoted by ovariectomy in this sedentary group. A recent study [89] also demonstrated high hemodynamic values of SBP and MAP in OVX sedentary mice and concluded that the depletion of E2 caused by ovariectomy enhanced angiotensin II-induced hypertension and associated pathophysiological changes, including impaired autonomic and renal function, increased water intake and urinary levels of vasopressin, renal hypertrophy, fibrosis, and ROS production. Another study in which ovariectomy was performed in spontaneously hypertensive rats (SHRs) also reported high SBP, DBP, and MAP values in the SHR-OVX sedentary group and demonstrated that ovariectomy decreases cardiac estrogen receptor ER-α and ER-β expression in hypertensive rats [90].

On the other hand, the OVX trained group (OVX-RT) presented lower hemodynamic values than did the OVX-SED group at the 13th week of evaluation, including SBP, DBP, MAP, and DP. Importantly, all of these hemodynamic values did not differ from the values obtained from the intact groups, including the sedentary (INT-SED) and trained groups (INT-RT), thus confirming the preventive effect of the RT protocol proposed in our study to prevent ovariectomy-induced arterial hypertension (AH). In this context, another important aspect to highlight is the independent effects of RT, at least in part, on renal mitochondrial function and blood pressure, considering the results on the intact rat group. A study carried out by Lino et al. [22], which also evaluated the effects of RT and OVX on systolic blood pressure, demonstrated other beneficial effects on vascular function (contraction of the smooth muscle) and histology (hypertrophic remodeling) of the aortic artery, which could suggest that other mechanisms may be involved in this issue.

Clinical and experimental studies have demonstrated that aerobic exercise and RT have favorable positive effects on hypertensive postmenopausal women and OVX rats, including improvements in blood pressure, cardiac function, and cardiorespiratory fitness, as well as protection against myocardial damage [22,41,91,92,93]. The use of exercise training as a nonpharmacological approach has been documented and strongly recommended for the management of AH [94]. A recent study revealed that concurrent exercise training (CET), a combination of aerobic exercise and RT, performed on OVX rats effectively prevented increases in the hemodynamic parameters (SBP, DBP, and MAP) that were observed in the OVX sedentary group [95]. Furthermore, when CET was associated with hydrochlorothiazide (HCTZ, an antihypertensive drug in the thiazide diuretic class), an additional adjustment in arterial pressure control mechanisms was achieved in hypertensive OVX rats, extending benefits to functional capacity and cardiovascular and autonomic control. The study concluded that exercise practices during medication treatment could directly contribute to minimizing the progression of AH-related mortality. All of the studies and arguments described above emphasize the importance of regular physical activity as a nonpharmacological tool for improving HA risk parameters in postmenopausal women and provide evidence that moderate-intensity and combined training (aerobic and resistance training) can significantly contribute to the prevention of hypertension in postmenopausal women [96].

Evidence from both human and animal studies indicates that low levels of physical activity can worsen CKD [97,98,99] and that a sedentary lifestyle could be both the cause and consequence of the progression of kidney disease [100]. Jeong et al. [101] conducted a study of sedentary individuals with CKD stages III and IV who completed a 12-week aerobic intervention (stationary bicycle three times per week). The results revealed a protective effect of exercise training on the progression of sympathetic nervous system (SNS) overactivity and increased aortic wave reflection associated with vascular stiffness, thus indicating that early initiation and continuous maintenance of an exercise training program may have greater clinical beneficial effects in CKD patients. Residues accumulated in CKD have inhibitory effects on the protein complexes of the respiratory chain, culminating in lower ATP synthesis [77]. Our results revealed that ovariectomy caused a significant decrease in the mRNA levels of CI and CIV, indicating a worse prognosis in the OVX-SED group. Correa et al. [102] conducted a study with 90 male and female hypertensive patients in stage 2 CKD who performed an RT protocol with blood flow restriction (RT-BFR) and without (RT) for six months and concluded that six months of RT with or without BFR decreases blood pressure and improves redox balance, nitric oxide (NO) bioavailability, vasoactive peptide levels, body composition, and muscle strength in patients with stage 2 CKD. In an animal model, Saud et al. [103] performed a study with a model of nephrectomy in male rats subjected to a RT protocol on a ladder for eight weeks before nephrectomy, beginning with 80% maximal carrying load to exhaustion, and subjected to RT for four weeks with 40% maximal carrying load after nephrectomy. This study revealed that the RT protocol prevents muscle loss and kidney injury in a very severe model of CKD. This beneficial outcome was especially associated with the group subjected to RT before and after nephrectomy (5/6Nx). The RT proposed in our study, which was similar to the protocol described by Saud et al. [103], had preventive effects against kidney mitochondrial dysfunction. The exercise regimen was able to improve mitochondrial bioenergetic function compared with the sedentary groups. OVX led to reduced gene expression related to the mitochondrial ETC and OXPHOS, whereas RT both prevented this reduction and increased gene expression in the trained groups. One of the limitations of our work was the failure to perform essential analyses of renal function, such as urine protein, creatinine clearance and cystatin C, which would allow us to discuss CKD caused by a decline of estrogen levels in OVX rats.

## 4. Materials and Methods

### 4.1. Ethical Approval

The experimental procedures were conducted in accordance with the principles outlined by the National Council of Animal Experimentation Control (Conselho Nacional de Controle de Experimentação Animal, CONCEA/Brazil), the Guide for the Care and Use of Laboratory Animals [104], and the Principles and Standards for Reporting Animal Experiments [105]. The study protocols were approved by the Committee on the Use of Experimental Animals at the Federal University of São Carlos (CEUA/UFSCar, letter approval 6331101218/2019: 11 March 2019).

### 4.2. Animals

Forty female offspring of adult Wistar rats (*Rattus norvergicus albinus*) were obtained from the breeding colony of the Federal University of São Carlos (UFSCar), São Carlos, Brazil. The animals were housed in collective cages with a constant temperature of 22 ± 2 °C and a 12 h light/dark cycle, with light starting at 07:00 AM. They had free access to water and chow and were fed Labina^®^ (a standard rat chow diet provided by Purina^®^, Descalvado, São Paulo, Brazil).

The rats arrived at the bioterium for adaptation, with an average weight of 165 ± 5 g. When they reached thirteen weeks old and approximately 271.53 ± 4.99 g of body mass, they were considered young adult rats [106,107], and ovariectomy (OVX) procedures were performed.

### 4.3. Experimental Groups

The animals were randomly assigned to four experimental groups (n = 40): intact sedentary (INT-SED, n = 10), ovariectomized sedentary group (OVX-SED, n = 10), intact resistance training group (INT-RT, n = 10), and ovariectomized resistance training group (OVX-RT, n = 10).

### 4.4. Ovariectomy (OVX)

This procedure involves bilateral removal of the ovaries, oviducts, and uterine horn tips in healthy reproductive-aged animals [18]. One to two weeks after ovariectomy, detecting the presence of 17β-estradiol in blood plasma is nearly impossible [108]. The animals were anesthetized with a ketamine–xylazine mixture (61.5–7.6 mg/100 g). Postsurgery, they received analgesic treatment with 2 mg/100 g tramadol hydrochloride and were treated with antibiotics (penicillin–streptomycin, 5 mg/kg) for 3 days. The animals underwent a 10-day recovery period before starting the resistance training (RT) protocol.

### 4.5. Resistance Training (RT) Protocol

The RT protocol was conducted over thirteen weeks and was adapted from Hornberger and Farrar [109]. The protocol involved a vertical ladder (1.1 m × 0.18 m) and a housing chamber (20 cm^3^) where the rats rested. The apparatus included a 50 mL conical tube with a fishing sinker attached to the proximal portion of the rat’s tail, and a self-adhesive foam was used to provide workload. The rats underwent three familiarization sessions with the protocol. They were considered fully adapted when they could ascend the vertical ladder three consecutive times, from the bottom to the housing chamber, with two minutes of rest in the chamber between climbs, without any external stimulation. During the familiarization sessions, the load apparatus was attached to their tails without added weight.

The initial maximum workload was determined by having the rats climb with a load equivalent to 75% of their body mass. This was followed by incremental increases of 30 g until the load impeded their ability to climb the entire length of the ladder. The highest load that the rats could carry across the entire length of the ladder was recorded as their maximum load capacity for that week’s training session. Subsequent maximum workload assessments were conducted weekly (on Mondays) and began with 65% of the maximum workload from the previous week. RT sessions were held twice a week (on Wednesdays and Fridays), each consisting of five climbs with loads of 65%, 70%, 75%, 80%, and 85% of the rat’s maximum workload for that week.

From the maximum workload (MW), the absolute maximum workload (AMW) and the relative maximum workload (RMW) were calculated via the following formulas: (a) AMW = MW + BM; (b) RMW = (MW + BM)/BM, where BM is body mass (grams).

### 4.6. Blood Pressure and Heart Rate Measurements

Systolic blood pressure (SBP), diastolic blood pressure (DBP), mean arterial pressure (MAP), and heart rate (HR) were measured via noninvasive tail plethysmography with a volume–pressure recording (VPR) system utilizing CODA^®^ equipment (Noninvasive Blood Pressure System, Kent Scientific Corporation, Torrington, CT, USA). The American Heart Association endorses this indirect method, also known as the VPR tail-cuff, for obtaining repeated blood pressure measurements in animals and for experimental designs involving a larger number of animals distributed in groups. The accuracy of this method has been validated by the study of Feng et al. [110]. To ensure precise measurements, the average of eight consecutive readings was used.

### 4.7. Euthanasia and Tissue Dissection

Forty-eight hours after the final RT session, the rats were euthanized via guillotine decapitation under mild inhalation sedation with isoflurane (1.05% of the minimum alveolar concentration in O_2_). The left kidney was rapidly dissected and divided into two portions: the first portion consisted of two small samples (4–8 mg) of the renal cortex, which were placed in a glass beaker containing ice-cold relaxing and biopsy preservation solution (BIOPS); the second portion, a larger sample including both the cortex and medulla, was frozen in liquid nitrogen and stored at −80 °C. The uterus was dissected and weighed, and its mass was normalized based on the tibial length (T) to calculate the uterus mass/tibia length ratio (U/T ratio).

### 4.8. Analysis of Mitochondrial Respiratory Function (High-Resolution Respirometry)

#### 4.8.1. Tissue Preparation, Mechanical and Chemical Permeabilization

The two small samples were stored in BIOPS solution (10 mM Ca-EGTA buffer, 0.1 µM free calcium, 20 mM imidazole, 20 mM taurine, 50 mM K-MES, 0.5 mM DTT, 6.56 mM MgCl_2_, 5.77 mM ATP, and 15 mM phosphocreatine, pH 7.1) for 5 min [111]. The renal cortex bundles were then separated via two pairs of very sharp forceps in a small Petri dish on ice. The preparation of 4–8 mg wet renal cortex samples required approximately 5 min. After complete mechanical permeabilization, the bundles were quickly transferred into a glass beaker containing 2 mL of ice-cold BIOPS solution with 20 µL of saponin stock solution (5 mg/mL, final concentration 50 µg/mL). The samples were gently agitated on ice for 30 min to achieve chemical permeabilization. The samples were subsequently transferred from the saponin solution into 2 mL of mitochondrial respiratory solution number 05 (MiR05) and shaken for 10 min on ice [49].

#### 4.8.2. Measurement of Oxygen Consumption

Oxygen consumption was measured via high-resolution respirometry (HRR) at 37 °C. The oxygen concentration (nmol/mL) and oxygen flux (pmol/[s·mg]), defined as the negative time derivative of the oxygen concentration divided by the kidney mass per volume, were recorded via DatLab software (version 7, Oroboros^®^, Innsbruck, Austria). The samples were placed in separate chambers (A and B) containing 2 mL of MiR05 (0.5 mmol/L EGTA, 3 mmol/L MgCl_2_, 60 mmol/L lactobionic acid, 20 mmol/L taurine, 10 mmol/L KH_2_PO_4_, 20 mmol/L HEPES, 110 mmol/L sucrose, and 1 g/L BSA, pH 7.0). The titration protocol, adapted from Kuznetsov et al. [112], included the following steps: (1) addition of 10 mmol/mL glutamate (Glu) and 5 mmol/mL malate (Mal) to all chambers to stimulate resting respiration or state 2 respiration; (2) addition of 1 mmol/mL ADP solution to stimulate oxygen consumption by complex I substrates (coupled or state 3 respiration); (3) addition of 10 µmol/mL cytochrome c (CytC) to test the integrity of the mitochondrial outer membrane [113]; (4) addition of 1 µmol/mL oligomycin (Oligo) to inhibit ATP synthase and induce state 4 respiration (leak respiration); and (5) addition of 2 µmol/mL carbonyl cyanide p-(trifluoromethoxy) phenylhydrazone (FCCP) to obtain an experimentally noncoupled respiratory state for evaluation of the respiratory capacity through the electron transfer system (ETS capacity) [59,114].

### 4.9. Citrate Synthase Activity

Citrate synthase (EC 2.3.3.1) catalyzes the reaction between acetyl coenzyme A and oxaloacetic acid to form citric acid and coenzyme A (CoA-SH). The activity of this enzyme was assessed via a colorimetric method that measures absorbance at 412 nm, which is attributable to the presence of thionitrobenzoic acid (TNB). TNB is formed through the reaction between 5,5′-dithiobis (2-nitrobenzoic acid) (DTNB) and CoA-SH [115]. Briefly, approximately 20 mg of renal cortex from each animal was mechanically homogenized in 20 µL/mg of tissue in buffer composed of 10 mM Tris-HCl (pH 7.4), 1 mM EDTA, and 0.1% (*v*/*v*) Triton X-100. The homogenate was centrifuged at 2000× *g* for 3 min, and 5 μL of the supernatant was used for the reactions in 195 μL of a solution containing 10 mM Tris-HCl (pH = 8.0), 50 μM acetyl-CoA, 250 μM oxaloacetic acid, and 100 µM DTNB. Reactions were conducted in triplicate and incubated at 37 °C for 20 min. The absorbance at 412 nm was recorded every 40 s via a microplate reader (Power Wave XS-2, Biotek Instruments, Winooski, VT, USA).

### 4.10. Gene Expression

Gene expression analysis was conducted by quantifying RNA via reverse transcription real-time polymerase chain reaction (RT–qPCR). The experiment adhered to the guidelines outlined in the Minimum Information for Publication of Quantitative Real-Time PCR Experiments (MIQE) [116].

#### 4.10.1. Extraction, Purification, Quantification, and Integrity of Total RNA

Fifty milligrams of renal cortex tissue were homogenized in 1 mL of TRI Reagent^®^ (Sigma, St. Louis, MO, USA) via a FastPrep^®^-24 Biomedicals instrument. RNA extraction and purification were carried out according to the TRI Reagent Protocol. The quantitative analysis was performed via spectrophotometry with a NanoDrop 2000 (Thermo Scientific™, Waltham, MA, USA), and the absorbance was measured at 260 nm. RNA integrity was assessed by visualizing the 28S and 18S ribosomal RNA bands under ultraviolet light via 1.2% agarose gel electrophoresis with ethidium bromide.

#### 4.10.2. Reverse Transcription to Complementary DNA (cDNA)

To eliminate potential contamination with genomic DNA, RNA samples (2 μg) were treated with amplification-grade DNase I (Invitrogen Corporation, Waltham, MA, USA) in accordance with the manufacturer’s specifications. The treated RNA was then used as a template in a qPCR reaction to confirm the effectiveness of the DNAse I treatment. The RNA was subsequently reverse transcribed into complementary DNA (cDNA) via Oligo dT (Exxtend, Paulínia, Brazil) and high-capacity cDNA reverse transcription kits (Applied Biosystems—Thermo Fisher Scientific, Waltham, MA, USA) in accordance with the manufacturer’s protocol.

#### 4.10.3. Real-Time Polymerase Chain Reaction (qPCR)

mRNA from the experimental samples was quantified via 0.1 mL MicroAmp^®^ Fast 96-well plates (Applied Biosystems^®^). Each well contained 3 µL of primer mixture, which was previously optimized for concentration and efficiency, 3 µL of cDNA (prepared from 1 µg of total RNA), and 6 µL of GoTaq^®^ qPCR Master Mix (Promega, Madison, WI, USA). The samples were analyzed via the QuantStudio™ 6 Flex real-time PCR detection system (Applied Biosystems). The thermal cycling conditions were as follows: an initial step at 95 °C for 10 min, followed by 40 cycles of 95 °C for 15 s and 60 °C for 1 min. Melting curve analysis was performed by increasing the temperature to 95 °C for 15 s, increasing it to 60 °C for 1 min, and then increasing it to 95 °C for 15 s. The samples were amplified in duplicate, and negative controls (no-template controls, NTC) enriched with sterile water were included in all of the analyses.

#### 4.10.4. Quality Control, Primer Concentration, and Efficiency

Gene targets and reference genes were selected via the National Center for Biotechnology Information (NCBI). For each ETC, only one gene was chosen for evaluation. Reference genes for endogenous controls were selected via geNorm software (version 3) (Table 3) [117]. The primer concentrations were optimized prior to generating efficiency curves, with primer efficiencies ranging from 90% to 110%. All primers were obtained from Exxtend Company (Paulínia, Brazil), and their sequences are listed in Table 4. To increase the robustness of the data, two reference genes were used for normalization, although only one gene is shown in the results.

### 4.11. Statistical Analysis

Statistical analyses were performed using Statistica 7.0 and GraphPad Prisma 8.2.1 software. The normality of the data was assessed with the Kolmogorov–Smirnov and Shapiro–Wilk tests. High-resolution respirometry, gene expression, and citrate synthase activity were analyzed via one-way ANOVA with repeated measures to evaluate the time effect. Fisher’s post hoc test was used for pairwise comparisons when a significant main effect was detected. Two-way ANOVA with Sidak’s multiple comparisons test was employed for comparisons of the maximal workload evaluations (MW, AMW, and RMW) and hemodynamic parameters (dependent variables) with time and groups (independent variables). A *p* value of <0.05 was considered statistically significant. The data are presented as the means ± standard deviations (SDs).

## 5. Conclusions

In the reduction of 17β-estradiol (E2) promoted by ovariectomy, there is a significant decrease in the OXPHOS capacity and electron transfer system capacity of sedentary animals, which are harmful conditions for mitochondrial kidney cortex function. These conditions were prevented by the RT adopted. There was also a change in the expression of genes related to the mitochondrial electron transfer chain and OXPHOS system, with decreases in the mRNA levels of Ant1 and subunits of respiratory complexes I and IV.

RT was able to increase the expression of the aforementioned genes, thus reversing the condition determined by ovariectomy and improving the expression of other genes directly related to the increased potential of CKD. The results of this study show the benefits of RT for the OVX group and the INT group.

## Figures and Tables

**Table 1:** Body parameters, body mass and tissue masses.

	Initial BM (g)	Final BM (g)	Left Kidney (g)	Uterine Mass (g)	Tibia Length (cm)	U/T Ratio
INT-SED	280.16 ± 4.21	314.17 ± 3.75 *	1.090 ± 0.30	0.570 ± 0.40 *^$^	3.95 ± 0.004	0.144 ± 0.03 *^$^
INT-RT	260.22 ± 5.34	291.68 ± 6.83 *^$#^	0.950 ± 0.16	0.680 ± 0.30 *^$^	3.96 ± 0.007	0.171 ± 0.02 *^$^
OVX-SED	270.95 ± 5.51	346.67 ± 5.56	0.957 ± 0.16	0.140 ± 0.13	3.94 ± 0.003	0.035 ± 0.02
OVX-RT	274.79 ± 4.89	341.10 ± 10.09	1.005 ± 0.21	0.150 ± 0.10	3.95 ± 0.005	0.038 ± 0.01

The groups were as follows: intact (INT), including intact sedentary (INT-SED) and intact resistance training (INT-RT); ovariectomy (OVX), including ovariectomy sedentary (OVX-SED) and ovariectomy resistance training (OVX-RT). Values are expressed as mean ± standard deviations (SD), with n = 10 per group. Symbols with superscripts indicate statistically significant differences between groups, as determined by one-way ANOVA followed by post hoc Fisher’s test, *p* < 0.05. * denotes a significant difference compared with the OVX-SED group; $ denotes a significant difference compared with the OVX-RT group; # denotes a significant difference compared with the INT-SED group.

**Table 2:** Evaluation of Systolic Blood Pressure, Heart Rate, and Double Product.

	EXPERIMENTAL GROUPS											
	INT-SED			INT-RT			OVX-SED			OVX-RT		
Time	SBP	HR	DP	SBP	HR	DP	SBP	HR	DP	SBP	HR	DP
PrOp	133.7 ± 4.06	490.33 ± 18.42	66,414.5 ± 3316.93	135.22 ± 3.95	440.71 ± 23.19	61,234.29 ± 4731.12	136.2 ± 2.12	474.8 ± 7.85	64,713.2 ± 2530.21	132.44 ± 3.5	466.62 ± 18.99	62,945.25 ± 3449
W1	136.5 ± 4.05	446 ± 12.37	61,103.2 ± 3001.87	134.44 ± 5.04	453.75 ± 20.35 ^c^	58,320.89 ± 2968.64	145.3 ± 6.43	453.44 ± 17.94	66,469.78 ± 4332.45	136.55 ± 6.42	434.77 ± 17.24	60,291.38 ± 2993.11
W5	141.5 ± 5.54	438.2 ± 12.41	62,307.7 ± 3661.55	148.44 ± 5.29	398.88 ± 7.23	59,421.56 ± 2934.23	147.8 ± 4.86	408.5 ± 13.65	60,118.8 ± 2028.86	149.66 ± 5.92	372.11 ± 40.68	55,940.89 ± 6619.52
W9	143.2 ± 4.85	416.9 ± 7.42 **^c^**	59,868.7 * ± 2734.73	147.88 ± 5.29	395.11 ± 9.26	58,717.56 * ± 3161.71	158.9 ± 4.59	436.55 ± 13.35 ^a,b,c^	70,406.44 ± 2398.45 ^c^	142.11 ± 4.16	405.55 ± 10.27	57,919.56 * ± 3037.74
W13	145.6 ± 4.35 *^,a^	401.7 ± 16.3 ^a,b,c,d^	58,788.5 ± 3648.36 *^,a^	148.22 ± 4.21 *^,a,b^	387.66 ± 9.2 ^a,b^	57,565.44 ± 2460.72 *	177.3 ± 3.54 ^a,b,c,d^	424.7 ± 14.79	73,034 ± 3266.77 ^c^	145.11 ± 2.64 *	405.44 ± 16.89	58,689.11 ± 2222.66 *

SBP: systolic blood pressure; HR: heart rate; DP: double product. Intact group (INT): intact sedentary (INT-SED) and intact resistance training (INT-RT). Ovariectomy group (OVX): ovariectomy sedentary (OVX-SED) and ovariectomy resistance training (OVX-RT). Values are expressed as the mean ± standard deviation (SD), n = 10 per group. To compare differences in systolic blood pressure (SBP), heart rate (HR) and double product (DP) between groups in the same week, and between weeks (time) in the same group, two-way ANOVA and post hoc Sidak’s multiple comparisons test were used, *p* < 0.05. * denotes a significant difference compared with the OVX-SED group; ^a^ denotes a significant difference compared with the initial evaluation (PRE); ^b^ denotes a significant difference compared with week 1; ^c^ denotes a significant difference compared with week 5; ^d^ denotes a significant difference compared with week 9.

**Table 3:** Sequences of endogenous primers selected via the geNorm program.

Gene	Primer Sequence	Concentration	Efficiency (%)
*B2m*	F: CGAGACCGATGTATATGCTTGC	100 nM	102.99
	R: CCGGATCTGGAGTTAAACTGG	100 nM	
*Psmc4*	F: TCGAGAAAGCATACAAGACCG	150 nM	102.63
	R: TCCTGGGTAAAGAGAAAACTAGC	150 nM	

**Table 4:** Target primer sequences.

Gene	Primer Sequence	Concentration	Efficiency (%)
*CI* (*Ndufb4*)	F: CGGCTTAAACGGGAGTATCTG	150 nM	100.83
	R: AAAGTGAGTTCTTGGGAGTGG	150 nM	
*CII* (*Sdha*)	F: TGTAAGAACATCAGAGCTGCG	150 nM	96.18
	R: CCCCTGTCAAACGTCTTCAG	150 nM	
*CIII* (*Uqcrc1*)	F: CCTTCAACATCTCCTACTCTGAG	150 nM	99.70
	R: TTTTGCCCCGAGTCACC	150 nM	
*CIV* *(Cox4i1)*	F: TTCGCTGAGATGAACAAGGG	150 nM	94.40
	R: GATCAAAGGTATGAGGGATGGG	150 nM	
*Atps* *(Atp5f1a)*	F: ATGTGGGCTTGTCTGTGTC	150 nM	98.07
	R: AGCATCCAGATCAGAACCAAAC	150 nM	
*Pic* *(Slc25a3)*	F: TCTACTTCAGGCTCCCTCG	150 nM	103.73
	R: TTCCTTTGCACTTTCAACACTG	150 nM	
*Ant1* *(Slc25a4)*	F: TTTCAGTGTCTCTGTGCAGG	150 nM	104.27
	R: GTCACACTCTGGGCAATCAT	150 nM	
*Cs*	F: AAGGAAAGGCTAAGAACCCC	150 nM	95.37
	R: ATTCATCTCCGTCATGCCATAG	300 nM	
*Ucp2*	F: GCCCCGAACCTTCTACAAG	300 nM	96.48
	R: ATTCATAGGCAGCCATCAGG	300 nM	

Comparative analysis of the results was performed via the 2^−ΔΔCt^ method [118].

## Data Availability

The data presented in this study are available on request from the corresponding author.

## References

[1] Nelson H.D. (2008). Menopause. Lancet.

[2] Vellanki K., Hou S. (2018). Menopause in CKD. Am. J. Kidney Dis..

[3] Suzuki H., Kondo K. (2012). Chronic kidney disease in postmenopausal women. Hypertens. Res..

[4] Mosca L., Appel L.J., Benjamin E.J., Berra K., Chandra-Strobos N., Fabunmi R.P., Grady D., Haan C.K., Hayes S.N., Judelson D.R. (2004). Evidence based guidelines for cardiovascular disease prevention in women. Arter. Thromb. Vasc. Biol..

[5] Rosano G.M., Vitale C., Marazzi G., Volterrani M. (2007). Menopause and cardiovascular disease: The evidence. Climatereic.

[6] Gallagher J.C. (2007). Effect of early menopause on bone mineral density and fractures. Menopause.

[7] Honigberg M.C., Zekavat S.M., Aragam K., Finneran P., Klarin D., Bhatt D.L., Januzzi J.L., Scott N.S., Natarajan P. (2019). Association of premature natural and surgical menopause with incident cardiovascular disease. JAMA.

[8] Anagnostis P., Christou K., Artzouchaltzi A.M., Gkekas N.K., Kosmidou N., Siolos P., Paschou S.A., Potoupnis M., Kenanidis E., Tsiridis E. (2019). Early menopause and premature ovarian insufficiency are associated with increased risk of type 2 diabetes: A systematic review and meta-analysis. Eur. J. Endocrinol..

[9] Ahmed S.B., Ramesh S. (2016). Sex hormones in women with kidney disease. Nephrol. Dial. Transplant..

[10] Qian D., Wang Z., Cheng Y., Luo R., Ge S.W., Xu G. (2022). Early Menopause May Associate with a Higher Risk of CKD and All-Cause Mortality in Postmenopausal Women: An Analysis of NHANES, 1999–2014. Front. Med..

[11] Hill N.R., Fatoba S.T., Oke J.L., Hirst J.A., O’Callaghan C.A., Lasserson D.S., Hobbs F.D.R. (2016). Global Prevalence of Chronic Kidney Disease—A Systematic Review and Meta-Analysis. PLoS ONE.

[12] van der Velde M., Matsushita K., Coresh J., Astor B.C., Woodward M., Levey A.S., Jong P.E., Gansevoort R.T. (2011). Lower estimated glomerular filtration rate and higher albuminuria are associated with all-cause and cardiovascular mortality. A collaborative meta-analysis of high risk population cohorts. Kidney Int..

[13] Yerram P., Karuparthi P.R., Hesemann L., Horst J., Whaley-Connell A. (2007). Chronic kidney disease and cardiovascular risk. J. Am. Soc. Hypertens..

[14] Tedla F.M., Brar A., Browne R., Brown C. (2011). Hypertension in Chronic KidneyDisease: Navigating the Evidence. Int. J. Hypertens..

[15] Burnier M., Damianaki A. (2023). Hypertension as Cardiovascular Risk Factor in Chronic Kidney Disease. Circ. Res..

[16] Ritz E. (2010). Hypertension and kidney disease. Clin. Nephrol..

[17] Mahmoodi B.K., Matsushita K., Woodward M., Blankestijn P.J., Cirillo M., Ohkubo T., Rossing P., Sarnak M.J., Stengel B., Yamagishiet K. (2012). Associations of kidney disease measures with mortality and end-stage renal disease in individuals with and without hypertension: A meta-analysis. Lancet.

[18] Koebele S.V., Bimonte-Nelson H.A. (2016). Modeling menopause: The utility of rodents in translational behavioral endocrinology research. Maturitas.

[19] Medina-Contreras J.M.L., Villalobos-Molina R., Zarain-Herzberg A., Balderas-Villalobos J. (2020). Ovariectomized rodents as a menopausal metabolic syndrome model. Mol. Cell. Biochem..

[20] Zhang L., Zhou M., Fang G., Tang Y., Chen Z., Liu X. (2013). Hypocholesterolemic effect of capsaicinoids by increased bile acids excretion in ovariectomized rats. Mol. Nutr. Food Res..

[21] Sivasinprasasn S., Sa-Nguanmoo P., Pratchayasakul W., Kumfu S., Chattipakorn S.C., Chattipakorn N. (2015). Obese-insulin resistance accelerates and aggravates cardiometabolic disorders and cardiac mitochondrial dysfunction in estrogen-deprived female rats. Age.

[22] Lino A.D.S., Vianna D., Oishi J.C., Souza M.V.C., Ruffoni L.D., Marin C.T., Avó L.R.S., Perez S.E.A., Rodrigues G.J., Tirapegui J. (2018). Resistance training and caloric restriction prevent systolic blood pressure rise by improving the nitric oxide effect on smooth muscle and morphological changes in the aorta of ovariectomized rats. PLoS ONE.

[23] Hassan H.A., Abdel-Wahhab M.A. (2012). Effect of soybean oil on atherogenic metabolic risks associated with estrogen deficiency in ovariectomized rats: Dietary soybean oil modulates atherogenic risks in ovariectomized rats. J. Physiol. Biochem..

[24] Shiguemoto G.E., Prestes J., Leite R.D., Pereira G.B., Pontes C.L.S., D’Ávila F.V., Botero J.P., Baldissera V., Nonaka K.O., Selistre-de-Araújo H.S. (2012). Effects of resistance training on matrix metalloproteinase-2 activity and biomechanical and physical properties of bone in ovariectomized and intact rats. Scand. J. Med. Sci. Sports.

[25] Yousefzadeh N., Jeddi S., Zarkesh M., Norouzirad R., Kashfi K., Ghasemi A. (2023). Protective effects of long-term nitrate administration against ovariectomy-induced kidney dysfunction in rats. Pharmacol. Rep..

[26] Ikeda M., Swide T., Vayl A., Lahm T., Anderson S., Hutchens M.P. (2015). Estrogen administered after cardiac arrest and cardiopulmonary resuscitation ameliorates acute kidney injury in a sex-and age-specific manner. Crit. Care.

[27] Pan J.S., Sheikh-Hamad D. (2019). Mitochondrial dysfunction in acute kidney injury and sex-specific implications. Med. Res. Arch..

[28] Patil N.K., Parajuli N., MacMillan-Crow L.A., Mayeux P.R. (2014). Inactivation of renal mitochondrial respiratory complexes and manganese superoxide dismutase during sepsis: Mitochondria-targeted antioxidant mitigates injury. Am. J. Physiol. Ren. Physiol..

[29] Galvan D.L., Green N.H., Danesh F.R. (2017). The hallmarks of mitochondrial dysfunction in chronic kidney disease. Kidney Int..

[30] Granata S., Zaza G., Simone S., Villani G., Latorre D., Pontrelli P., Carella M., Schena F.P., Grandaliano G., Pertosa G. (2009). Mitochondrial dysregulation and oxidative stress in patients with chronic kidney disease. BMC Genom..

[31] Che R., Yuan Y., Huang S., Zhang A. (2014). Mitochondrial dysfunction in the pathophysiology of renal diseases. Am. J. Physiol. Ren. Physiol..

[32] Su M., Dhoopun A.R., Yuan Y., Huang S., Zhu C., Ding G., Liu B., Yang T., Zhang A. (2013). Mitochondrial dysfunction is an early event in aldosterone-induced podocyte injury. Am. J. Physiol. Ren. Physiol..

[33] Ventura-Clapier R., Piquereau J., Veksler V., Garnier A. (2019). Estrogens, Estrogen Receptors Effects on Cardiac and Skeletal Muscle Mitochondria. Front. Endocrinol..

[34] Yin L., Luo M., Wang R., Ye J., Wang X. (2021). Mitochondria in sex-hormone induced disorder of energy metabolism in males and females. Front. Endocrinol..

[35] Lin Y., Lee S. (2018). Cardiovascular Benefits of Exercise Training in Postmenopausal Hypertension. Int. J. Mol. Sci..

[36] Yuk J.S. (2024). Relationship between menopausal hormone therapy and breast cancer: A nationwide population-based cohort study. Int. J. Gynecol. Obstet..

[37] Manson J., Chlebowski R., Stefanick M., Aragaki A., Rossouw J., Prentice R., Anderson G.L., Howard B.V., Thomson C.A., Lacroix A.Z. (2013). Menopausal hormone therapy and health outcomes during the intervention and extended post stopping phases of the Women’s Health Initiative randomized trials. JAMA.

[38] Manson J., Aragaki K., Rossouw J., Anderson G., Prentice R., LaCroix A., Chlebowski R.T., Howard B.V., Thomson C.A., Margolis K.L. (2017). Menopausal hormone therapy and long term all-cause and cause-specific mortality: The Women’s Health Initiative randomized trials. JAMA.

[39] Stojanovska L., Apostolopoulos V., Polman R., Borkoles E. (2014). To exercise, or, not to exercise during menopause and beyond. Maturitas.

[40] Grindler N.M., Santoro N.F. (2015). Menopause and exercise. Menopause J. N. Am. Menopause Soc..

[41] Figueroa A., Park S.Y., Seo D.Y., Sanchez-Gonzalez M.A., Baek Y.H. (2011). Combined resistance and endurance exercise training improves arterial stiffness, blood pressure, and muscle strength in postmenopausal women. Menopause.

[42] Marin C.T., Lino A.D.S., Avelar I.D.S., Barbosa M.R., Scarlato G.C.G., Cavalini D.F., Tamanini F., Alexandrino A.V., Vercesi A.E., Shiguemoto G.E. (2023). Resistance training prevents dynamics and mitochondrial respiratory dysfunction in vastus lateralis muscle of ovariectomized rats. Exp. Gerontol..

[43] Moinuddin I., Leehey D.J. (2008). A Comparison of Aerobic Exercise and Resistance Training in Patients with and without Chronic Kidney Disease. Adv. Chronic Kidney Dis..

[44] Malheiro L.F.L., Fernandes M.M., Oliveira C.A., Barcelos I.S., Fernandes A.J.V., Silva B.S., Ávila J.S., Soares T.J., Amaral L.S.B. (2024). Renoprotective mechanisms of exercise training against acute and chronic renal diseases—A perspective based on experimental studies. Life Sci..

[45] Leite A.B., Lima H.N., Flores C.O., Oliveira C.A., Cunha L.E.C., Neves J.L., Correia T.M.L., de Melo F.F., Oliveira M.V., de Magalhães A.C.M. (2021). High-intensity interval training is more effective than continuous training to reduce inflammation markers in female rats with cisplatin nephrotoxicity. Life Sci..

[46] Amaral L.S.B., Silva F.A., Correia V.B., Andrade C.E., Dutra B.A., Oliveira M.V., de Magalhães A.C., Volpini R.A., Seguro A.C., Coimbra T.M. (2017). Beneficial effects of previous exercise training on renal changes in streptozotocin-induced diabetic female rats. Exp. Biol. Med..

[47] Zeynali F., Nematbakhsh M., Mojtahedi H., Poorshahnazari A., Talebi A., Pezeshki Z., Mazaheri S., Moslemi F. (2015). Protective role of aerobic exercise against cisplatin-induced nephrotoxicity in rats. Asian. J. Sports Med..

[48] Rodrigues A.M., Bergamaschi C.T., Araújo R.C., Mouro M.G., Rosa T.S., Higa E.M.S. (2011). Effects of training and nitric oxide on diabetic nephropathy progression in type I diabetic rats. Exp. Biol. Med..

[49] Yamakoshi S., Nakamura T., Mori N., Suda C., Kohzuki M., Ito O. (2021). Effects of exercise training on renal interstitial fibrosis and renin-angiotensin system in rats with chronic renal failure. J. Hypertens..

[50] Almeida A.A., Correia T.M.L., Pires R.A., Silva D.A., Coqueiro R.S., Machado M., de Magalhães A., Queiroz R., Soares T., Pereira R. (2022). Nephroprotective effect of exercise training in cisplatin-induced renal damage in mice: Influence of training protocol. Braz. J. Med. Biol. Res..

[51] Oliveira C., Mercês É., Portela F., Benedictis J., Benedictis L., Silva A., Campanati J.d.A.G., Freire de Melo F., Oliveira M.V., Mendes de Magalhães A.C. (2023). Benefits of high-intensity interval training compared to continuous training to reduce apoptotic markers in female rats with cisplatin nephrotoxicity—Possible modulatory role of IL-11. Apoptosis.

[52] Bayod S., Del Valle J., Lalanza J., Sanchez-Roige S., de Luxan-Delgado B., Coto-Montes A., Canudas A., Camins A., Escorihuela R., Pallàs M. (2012). Long-term physical exercise induces changes in sirtuin 1 pathway and oxidative parameters in adult rat tissues. Exp. Gerontol..

[53] He W., Wang Y., Zhang M.Z., You L., Davis L.S., Fan H., Yang H.-C., Fogo A.B., Zent R., Harris R.C. (2010). Sirt1 activation protects the mouse renal medulla from oxidative injury. J. Clin. Investig..

[54] Dong Y.J., Liu N., Xiao Z., Sun T., Wu S.H., Sun W.X., Xu Z.-G., Yuan H. (2014). Renal protective effect of sirtuin 1. J. Diabetes Res..

[55] Klinge C.M. (2020). Estrogenic control of mitochondrial function. Redox Biol..

[56] Anagnostis P., Theocharis P., Lallas K., Konstantis G., Mastrogiannis K., Bosdou J.K., Lambrinoudaki I., Stevenson J.C., Goulis D. (2020). Early menopause is associated with increased risk of arterial hypertension: A systematic review and meta-analysis. Maturitas.

[57] Brand M.D., Nicholls D.G. (2011). Assessing mitochondrial dysfunction in cells. Biochem. J..

[58] Park S.Y., Gifford J.R., Andtabacha R.H.I., Trinity J.D., Hyngstrom J.R., Garten R.S., Diakos N.A., Ives S.J., Dela F., Larsen S. (2014). Cardiac, skeletal, and smooth muscle mitochondrial respiration: Are all mitochondria created equal?. Am. J. Physiol. Heart Circ. Physiol..

[59] Gnaiger E. (2014). Mitochondrial Pathways and Respiratory Control. An Introduction to OXPHOS Analysis, 4th ed., Mitochondr Physiol Network 17.18. OROBOROS MiPNet Publications, Innsbruck. https://wiki.oroboros.at/images/f/fc/Gnaiger_2014_Mitochondr_Physiol_Network_MitoPathways.pdf.

[60] Gnaiger E. (2009). Capacity of oxidative phosphorylation in human skeletal muscle: New perspectives of mitochondrial physiology. Int. J. Biochem. Cell Biol..

[61] Ruas S.R., Siqueira-Campos E.S., Rodrigues-Silva E., Castilho R.F. (2018). High glycolytic activity of tumor cells leads to underestimation of electron transport system capacity when mitochondrial ATP synthase is inhibited. Sci. Rep..

[62] Jedlička J., Grundmanová M., Švíglerová J., Tůma Z., Nalos L., Rajdl D., Štengl M., Kuncová J. (2022). Mitochondrial Dysfunction in Kidney Cortex and Medulla of Subtotally Nephrectomized Rats. Physiol. Res..

[63] Porter C., Reidy P.T., Bhattarai N., Sidossis L.S., Rasmussen B.B. (2015). Resistance exercise training alters mitochondrial function in human skeletal muscle. Med. Sci. Sports Exerc..

[64] Jacobs R.A., Lundby C. (2013). Mitochondria express enhanced quality as well as quantity in association with aerobic fitness across recreationally active individuals up to elite athletes. J. Appl. Physiol..

[65] Hütter E., Unterluggauer H., Garedew A., Jansen-Dürr P., Gnaiger E. (2006). High-resolution respirometry–a modern tool in aging research. Exp. Gerontol..

[66] Divakaruni A.S., Brand M.D. (2011). The regulation and physiology of mitochondrial proton leak. Physiology.

[67] Griffiths K.K., Wang A., Wang L., Tracey M., Kleiner G., Quinzii C.M., Sun L., Yang G., Perez-Zoghbi J.F., Licznerski P. (2020). Inefficient Thermogenic Mitochondrial Respiration Due to Futile Proton Leak in a Mouse Model of Fragile X Syndrome. FASEB J..

[68] Sreedhar A., Zhao Y. (2017). Uncoupling Protein 2 and Metabolic Diseases. Mitochondrion.

[69] Friederich M., Fasching A., Hansell P., Nordquist L., Palm F. (2008). Diabetes-induced up-regulation of uncoupling protein-2 results in increased mitochondrial uncoupling in kidney proximal tubular cells. Biochim. Biophys. Acta..

[70] Bhargava P., Schnellmann R.G. (2017). Mitochondrial energetics in the kidney. Nat. Rev. Nephrol..

[71] Wang Z., Ying Z., Bosy-Westphal A., Junyi Z., Britta S., Wiebke L., Heymsfield S.B., Manfred M. (2010). Specific metabolic rates of major organs and tissues across adulthood: Evaluation by mechanistic model of resting energy expenditure. Am. J. Clin. Nutr..

[72] Sharma K. (2017). Mitochondrial dysfunction in the diabetic kidney. Adv. Exp. Med. Biol..

[73] Sharma K. (2015). Mitochondrial hormesis and diabetic complications. Diabetes.

[74] Szeto H.H. (2017). Pharmacologic approaches to improve mitochondrial function in AKI and CKD. J. Am. Soc. Nephrol..

[75] Eirin A., Lerman A., Lerman L.O. (2018). Enhancing mitochondrial health to treat hypertension. Curr. Hypertens. Rep..

[76] McCrimmon A., Domondon M., Sultanova R.F., Ilatovskaya D.V., Stadler K. (2020). Comprehensive assessment of mitochondrial respiratory function in freshly isolated nephron segments. Am. J. Physiol. Ren. Physiol..

[77] Wang Y., Yang J., Zhang Y., Zhou J. (2024). Focus on Mitochondrial Respiratory Chain: Potential Therapeutic Target for Chronic Renal Failure. Int. J. Mol. Sci..

[78] Singh P. (2023). Reprogramming of Energy Metabolism in Kidney Disease. Nephron.

[79] Zhu Z., Hu J., Chen Z., Feng J., Yang X., Liang W., Ding G. (2022). Transition of acute kidney injury to chronic kidney disease: Role of metabolic reprogramming. Metab. Clin. Exp..

[80] Barrientos A. (2002). In vivo and in organello assessment of OXPHOS activities. Methods.

[81] Yubero D., Adin A., Montero R., Jou C., Jiménez-Mallebrera C., García-Cazorla A., Nascimento A., O’Callaghan M.M., Montoya J., Gortet L. (2016). A statistical algorithm showing coenzyme Q_10_ and citrate synthase as biomarkers for mitochondrial respiratory chain enzyme activities. Sci. Rep..

[82] Frankish B.P., Najdovska P., Xu H., Wette S.G., Murphy R.M. (2021). Effects of voluntary wheel running on mitochondrial content and dynamics in rat skeletal muscle. J. Muscle Res. Cell Motil..

[83] Ullian M.E., Gantt B.J., Ford A.K., Tholanikunnel B.G., Spicer E.K., Fitzgibbon W.R. (2003). Potential importance of glomerular citrate synthase activity in remnant nephropathy. Kidney Int..

[84] Mapuskar K.A., Wen H., Holanda D.G., Rastogi P., Steinbach E., Han R., Coleman M.C., Attanasio M., Riley D., Spitz D.R. (2019). Persistent increase in mitochondrial superoxide mediates cisplatin-induced chronic kidney disease. Redox Biol..

[85] Dines V.A., Garovic V.D. (2023). Menopause and chronic kidney disease. Nat. Rev. Nephrol..

[86] Matsushita K., Ballew S.H., Wang A.Y., Kalyesubula R., Schaeffner E., Agarwal R. (2022). Epidemiology and risk of cardiovascular disease in populations with chronic kidney disease. Nat. Rev. Nephrol..

[87] Rashid A., Jamil A., Khan Z., Shakoor M., Kamal U., Khan I., Akram A., Shahabi M., Yamani N., Ali S. (2024). Trends in mortality related to kidney failure and diabetes mellitus in the United States: A 1999–2020 analysis. J. Nephrol..

[88] Damianaki A., Polychronopoulou E., Wuerzner G., Burnier M. (2022). New Aspects in the Management of Hypertension in Patients with Chronic Kidney Disease not on Renal Replacement Therapy. High Blood Press. Cardiovasc. Prev..

[89] Dutta S.R., Singh P., Malik K.U. (2023). Ovariectomy Via 12/15-lipoxygenase Augments Angiotensin II-Induced Hypertension and Its Pathogenesis in Female Mice. Hypertension.

[90] Lin Y.Y., Hong Y., Zhou M.C., Huang H.L., Shyu W.C., Chen J.S., Ting H., Cheng Y.J., Yang A.L., Lee S.D. (2020). Exercise training attenuates cardiac inflammation and fibrosis in hypertensive ovariectomized rats. J. Appl. Physiol..

[91] Shimojo G.L., Palma R.K., Brito J.O., Sanches I.C., Irigoyen M.C., De Angelis K. (2015). Dynamic resistance training decreases sympathetic tone in hypertensive ovariectomized rats. Braz. J. Med. Biol. Res..

[92] Da Palma R.K., Moraes-Silva I.C., Dias S.D., Shimojo G.L., Conti F.F., Bernardes N., Barboza C.A., Sanches I.C., Araujo A.S., Irigoyen M.C. (2016). Resistance or aerobic training decreases blood pressure and improves cardiovascular autonomic control and oxidative stress in hypertensive menopausal rats. J. Appl. Physiol..

[93] Son W.M., Sung K.D., Cho J.M., Park S.Y. (2017). Combined exercise reduces arterial stiffness, blood pressure, and blood markers for cardiovascular risk in postmenopausal women with hypertension. Menopause.

[94] Whelton P.K., Carey R.M., Aronow W.S., Casey D.E., Collins K.J., Himmelfarb C.D., DePalma S.M., Gidding S., Jamerson K.A., Jones D.W. (2018). ACC/AHA/AAPA/ABC/ACPM/AGS/APhA/ASH/ASPC/NMA/PCNA Guideline for the Prevention, Detection, Evaluation, and Management of High Blood Pressure in Adults: A Report of the American College of Cardiology/American Heart Association Task Force on Clinical Practice Guidelines. Circulation.

[95] Ferreira M.J., Silva M.P.S.F., Dias D.S., Bernardes N., Irigoyen M.C., De Angelis K. (2023). Concurrent exercise training induces additional benefits to hydrochlorothiazide: Evidence for an improvement of autonomic control and oxidative stress in a model of hypertension and postmenopause. PLoS ONE.

[96] Tan A., Thomas R.L., Campbell M.D., Prior S.L., Bracken R.M., Churm R. (2023). Effects of exercise training on metabolic syndrome risk factors in post-menopausal women: A systematic review and meta-analysis of randomized controlled trials. Clin. Nutr..

[97] Luan X., Tian X., Zhang H., Huang R., Li N., Chen P., Wang R. (2019). Exercise as a prescription for patients with various diseases. J. Sport Health Sci..

[98] Mallamaci F., Pisano A., Triepi G. (2020). Physical activity in chronic kidney disease and the EXerCise Introduction to Enhance trial. Nephrol. Dial. Transplant..

[99] Wilkinson T.J., McAdams-DeMarcob M., Bennett P.N., Wilund K. (2020). Advances in exercise therapy in predialysis chronic kidney disease, hemodialysis, peritoneal dialysis, and kidney transplantation. Curr. Opin. Nephrol. Hypertens..

[100] Villanego F., Naranjo J., Vigara L.A., Cazorla J.M., Montero M.E., García T., Torrado J., Mazuecos A. (2020). Impacto del ejercicio físico en pacientes con enfermedad renal crónica: Revisión sistemática y metaanálisis. Nefrologia.

[101] Jeong J., Sprick J.D., DaCosta D.R., Mammino K., Nocera J.R., Park J. (2023). Exercise modulates sympathetic and vascular function in chronic kidney disease. Clin. Med..

[102] Corrêa H.L., Neves R.V.P., Deus L.A., Maia B.C.H., Maya A.T., Tzanno-Martins C., Souza M.K., Silva J.A.B., Haro A.S., Costa F. (2021). Low-load resistance training with blood flow restriction prevent renal function decline: The role of the redox balance, angiotensin 1–7 and vasopressin. Physiol. Behav..

[103] Saud A., Luiz R.S., Leite A.P.O., Muller C.R., Visona I., Reinecke N., Silva W.H., Gloria M.A., Razvickas C.V., Casarini D.E. (2021). Resistance exercise training ameliorates chronic kidney disease outcomes in a 5/6 nephrectomy model. Life Sci..

[104] National Research Council (2011). Guide for the Care and Use of Laboratory Animals.

[105] Grundy D. (2015). Principles and standards for reporting animal experiments in The Journal of Physiology and Experimental Physiology. J. Physiol..

[106] Horcajada M.N., Habauzit V., Trzeciakiewicz A., Morand C., Gil-Izquierdo A., Mardon J., Lebecque P., Davicco M.J., Chee W.S.S., Coxam V. (2008). Hesperidin inhibits ovariectomized-induced osteopenia and shows differential effects on bone mass and strength in young and adult intact rats. J. Appl. Physiol..

[107] Souza S.B.C., Flues K., Paulini J. (2007). Role of exercise training in cardiovascular autonomic dysfunction and mortality in diabetic ovariectomized rats. Hypertension.

[108] Brinton R.D. (2012). Minireview: Translational animal models of human menopause: Challenges and emerging opportunities. Endocrinology.

[109] Hornberger T.A., Farrar R.P. (2004). Physiological hypertrophy of the FHL muscle following 8 weeks of progressive resistance exercise in the rat. Can. J. Appl. Physiol..

[110] Feng M., Whitesall S., Zhang Y., Beibel M., D’ Alecy L., DiPetrillo K. (2008). Validation of Volume—Pressure Recording Tail-Cuff Blood Pressure Measurements. Am. J. Hypertens..

[111] Letellier T., Malgat M., Coquet M., Moretto B., Parrot-Roulaud F., Mazat J.P. (1992). Mitochondrial Myopathy Studies on Permeabilized Muscle Fibers 1. Pediatr. Res..

[112] Kuznetsov A.V., Veksler V., Gellerich F.N., Saks V., Margreiter R., Kunz W.S. (2008). Analysis of mitochondrial function in situ in permeabilized muscle fibers, tissues and cells. Nat. Protoc..

[113] Tepp K., Shevchuk I., Chekulayev V., Timohhina N., Kuznetsov A.V., Guzun R., Saks V., Kaambre T. (2011). High efficiency of energy flux controls within mitochondrial interactosome in cardiac intracellular energetic units. Biochim. Biophys. Acta.

[114] Chance B., Williams G.R. (1955). Respiratory enzymes in oxidative phosphorylation I. Kinetics of oxygen utilization. J. Biol. Chem..

[115] Shepherd D., Garland P.B. (1969). Citrate synthase from rat liver: [EC 4.1. 3.7 Citrate oxaloacetage-lyase (CoA-acetylating)]. Methods Enzymol..

[116] Bustin S.A., Benes V., Garson J.A., Hellemans J., Huggett J., Kubista M., Mueller R., Nolan T., Pfaffl M.W., Shipley G.L. (2009). The MIQE Guidelines: Minimum Information for Publication of Quantitative Real-Time PCR Experiments. Clin. Chem..

[117] Vandesompele J., De Preter K., Pattyn F., Poppe B., Van Roy N., De Paepe A., Speleman F. (2002). Accurate normalization of real-time quantitative RT-PCR data by geometric averaging of multiple internal control genes. Genome Biol..

[118] Livak N.J., Schmittgen T.D. (2001). Analysis of Relative Gene Expression Data Using Real-Time Quantitative PCR and the 2^−ΔΔ*C*^_T_ Method. Methods.

